# The Prognostic Significance of Metabolic Syndrome and a Related Six-lncRNA Signature in Esophageal Squamous Cell Carcinoma

**DOI:** 10.3389/fonc.2020.00061

**Published:** 2020-02-18

**Authors:** Yu Liu, Liyu Wang, Hengchang Liu, Chunxiang Li, Jie He

**Affiliations:** ^1^Department of Thoracic Surgery, National Cancer Center/National Clinical Research Center for Cancer/Cancer Hospital, Chinese Academy of Medical Sciences and Peking Union Medical College, Beijing, China; ^2^Department of Colorectal Surgery, National Cancer Center/National Clinical Research Center for Cancer/Cancer Hospital, Chinese Academy of Medical Sciences and Peking Union Medical College, Beijing, China

**Keywords:** metabolic syndrome, lncRNA signature, prognosis, esophageal squamous cell carcinoma, clinical predictive model

## Abstract

**Background:** Metabolic syndrome (MetS) is associated with the development of esophageal squamous cell carcinoma (ESCC), and long non-coding RNAs (lncRNAs) are involved in a variety of mechanisms of MetS and tumor. This study will explore the prognostic effect of MetS and the associated lncRNA signature on ESCC.

**Methods:** Our previous RNA-chip data (GSE53624, GSE53622) for 179 ESCC patients were reanalyzed according to MetS. The recurrence-free survival (RFS) was collected for these patients. The status of the MetS-related tumor microenvironment was analyzed with the CIBERSORT and ESTIMATE algorithms. A lncRNA signature was established with univariate and multivariate Cox proportional hazards regression (PHR) analysis and verified using the Kaplan–Meier survival curve analysis and time-dependent receiver operating characteristic (ROC) curves. A clinical predictive model was constructed based on multiple risk factors, evaluated using C-indexes and calibration curves, and verified using data from the GEO and TCGA databases.

**Results:** The results showed that MetS was an independent risk factor for ESCC patients conferring low OS and RFS. Tumor microenvironment analysis indicated that patients with MetS have high stromal scores and M2 macrophage infiltration. A six-lncRNA signature was established by 60 ESCC patients randomly selected from GSE53624 and identified with an effective predictive ability in validation cohorts (59 patients from GSE53624 and 60 patients from GSE53622), subgroup analysis, and ESCC patients from TCGA. MetS and the six-lncRNA signature could be regarded as independent risk factors and enhanced predictive ability in the clinical predictive model.

**Conclusions:** Our results indicated that MetS was associated with poor prognosis in ESCC patients, and the possible mechanism was related to changes in the tumor microenvironment. MetS and the six-lncRNA signature could also serve as independent risk factors with available clinical application value.

## Introduction

Metabolic syndrome (MetS) represents a cluster of metabolic disorders, including obesity, hyperglycemia, hypertension, and dyslipidemia. The prevalence of MetS remains stable in nearly 35% of all adults in the United States (1, 2). A recent study showed a high prevalence of MetS at 33.9% (31% in men and 36.8% in women), with a dramatic increase from 2000 to 2010 in China (3). MetS is an embodiment of comprehensive effects and is associated with multiple diseases, including tumors (4, 5). The presence of MetS can increase the risk and influence the prognosis of various tumors, such as colorectal cancer, breast cancer, and prostate cancer (6–8). The potential mechanisms mainly include obesity, chronic hyperinsulinemia, sustained inflammation-related signaling, estrogen signaling, and extracellular matrix remodeling (9, 10).

Esophageal cancer is the fourth most common cancer in China, with a 5-year survival rate of ~20%, because patients are often diagnosed at an advanced stage (11–13). Some studies have shown that obesity or MetS is associated with the risk and development of esophageal cancer (13–18). A prospective study of 2,396 esophageal squamous cell carcinoma (ESCC) patients demonstrated that MetS was a significant independent predictor of poor prognosis and was associated with glycolipid metabolism disorder (13). However, studies have also shown that MetS might be a good prognostic factor because of the good nutritional status of ESCC patients (15, 18).

Long non-coding RNAs (lncRNAs) are closely associated with tumor development and influence the prognosis of patients with tumors (19–21). The previous studies have indicated the predictive ability of lncRNA signatures for the prognosis of ESCC including our previous study (22–24). In addition, some studies also revealed that lncRNA was associated with the MetS or related metabolism disorder (25, 26). Considering the ambiguous relationship between MetS and survival of ESCC patients, we collected preoperative information of those 179 cases to identify a correlation. The relevant mechanism was explored with gene expression profiling, and a MetS-related lncRNA signature was established for predicting prognosis.

## Materials and Methods

### The Reanalysis of ESCC Patient Clinical Data

In total, 179 patients were surgically confirmed to have ESCC at the Cancer Institute and Hospital of the Chinese Academy of Medical Sciences (CAMS) between 2005 and 2008. Preoperative clinical information about MetS, including preoperative body mass index, fasting blood glucose level, blood pressure, and triglyceride and high-density lipoprotein cholesterol levels, was collected. We defined MetS in our study according to the criteria of the Chinese Diabetes Society in 2004 as previous studies shown (13). Recurrence-free survival (RFS) was collected and defined as the interval from the data of surgery to the end of follow-up results or death.

### The Selection of Esophageal Cancer and Related Cancer Databases

The microarrays or RNA-seq data were searched with keywords as esophagus/esophageal cancer or esophagus/esophageal carcinoma from public database. The inclusive criteria are as follows: (1) microarrays or RNA-seq database of ESCC and more than 10 patients' tumor tissue; (2) available clinical information, accurate follow-up, and valuable prognostic information; and (3) the database includes the lncRNA expression profiles.

The expression profiles for 119 patients from GSE53624 and 60 patients from GSE53622 were generated using the Agilent human lncRNA+mRNA array V.2.0 platform. The 119 ESCC patients from GSE53624 were randomly divided into 60 ESCC and 59 ESCC patients by caret package of R (set.seed = 1,000, *p* = 0.50). We used the 60 ESCC patients as the training cohorts, and 59 ESCC patients were used as the internal validation cohort. Another 60 ESCC patients from GSE53622 were used as an independent validation cohort (http://www.ncbi.nlm.nih.gov/geo) (22, 27). Meanwhile, the transcriptome expression profiles and corresponding clinical information of ESCC were downloaded from TCGA (https://tcga-data.nci.nih.gov/). All selected expression datasets were standardized before use.

### The Analysis of the Tumor Microenvironment by Immune Infiltration Profiles and Stromal Scores

The tumor and adjacent normal tissues of 179 ESCC patients (GSE53624, GSE53622) were used for analysis. CIBERSORT is a deconvolution algorithm that uses a signature matrix of 547 genes to represent 22 types of infiltrating immune cells (28). CIBERSORT derives a *p* value for the deconvolution for each sample using Monte Carlo sampling, and samples with *p* > 0.05 are removed. The ESTIMATE algorithm (29) was used to calculate the immune and stromal scores for each tumor sample. These algorithms were used to analyze the clinical correlation among MetS status, T stage, N stage, TNM stage, tumor grade, and survival.

### Identification of lncRNA Signature to Predict the Prognosis for ESCC Patients

Differences in mRNA and lncRNA expression (GSE53624, GSE53622) classified with MetS of 179 ESCC patients were screened by fold change > 1 and adj *p* < 0.05. Next, weighted gene co-expression network analysis (WGCNA) was also used to determine the relationship between the module gene and MetS. Significantly correlated module genes were selected using univariate Cox proportional hazards regression (PHR) analysis to calculate the correlation with OS and RFS. The identified lncRNAs with |*z*| > 2 were selected, and then, multivariate stepwise Cox PHR analysis was used to build a lncRNA predicting model and identify the best risk score cutoff value to distinguish ESCC patients. Kaplan–Meier survival curve analysis and log-rank tests were used to calculate differences between groups. Subsequently, time-dependent receiver operating characteristic (ROC) curves and areas under ROC curves (AUCs) were calculated to verify the reliability of our lncRNA signature.

### Validation of the Six-lncRNA Signature

Validation sets of 59 cases from GSE53624 and 60 patients from GSE53622 were used to validate the predictive power of our lncRNA signature. A total of 179 patients and 80 patients from TCGA were all used for analysis. The caret package of R was used randomly divided 179 ESCC patients into two groups to verify the predictive power (set.seed = 1,000, *p* = 0.50). Subgroup analyses of 179 ESCC patients by tumor grade and TNM stage were also used to evaluate the predictive power. The detailed workflow is shown in **Figure 9**.

### Functional Enrichment Analysis and Construction of a PPI Network

MetS-relevant module genes were selected by WGCNA and analyzed by DAVID (https://david.ncifcrf.gov/) (30). Meanwhile, a protein–protein interaction (PPI) network was constructed using the STRING database and Cytoscape software (31, 32). We selected the top 50 hub genes by cytoHubba and analyzed them using ClueGO in Cytoscape software.

### The Clinical Predictive Model

The MetS, six-lncRNA signature and 179 ESCC patients' risk factors were used to establish a clinical predictive model to analyze the application value. The performance of the nomogram was assessed using Harrel's concordance indexes (C-indexes) and calibration curves. Bootstraps with 1000 resamples were used for these activities. We used the random group as internal validation and ESCC patients as an external validation group.

### Statistical Analysis

Clinical statistical analysis was performed using EmpowerStats (http://www.empowerstats.com/). A *p* < 0.05 was considered statistically significant. Hazard ratios (HRs) and 95% confidence intervals (CIs) were also calculated. ActivePerl software (www.activestate.com/products/activeperl/) was used to extract and collate data. R software with packages edgeR, ggplot2, limma, e1071, parallel, estimate, impute, rms, foreign, survival, caret, ROC, and timeROC (Bioconductor.org/biocLite.R) was used for statistical analysis and plotting.

## Results

### Mets Is an Independent Prognostic Factor for ESCC

**Figure 1:**
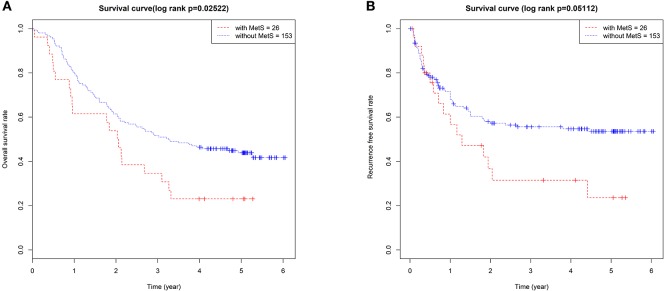
The MetS had a bad effect on the prognosis of ESCC. The Kaplan–Meier survival analysis of the OS **(A)** and RFS **(B)** between 179 ESCC patients with and without MetS.

The 179 ESCC patients were divided according to MetS. Twenty-six cases (14.5%) met the criteria of MetS, and 153 cases (85.5%) did not. We compared the clinical characteristics between the two groups, and significantly low overall survival (*p* = 0.025, Figure 1A) and RFS (*p* = 0.051, Figure 1B) were detected in patients with MetS. There were no differences in sex, TNM stage, or other clinical characteristics (Table 1).

**Table 1:** The clinical characteristics at baseline, stratified by metabolic syndrome (MetS).

**Variable**	**Without MetS**	**With MetS**	***p* value**
No. of patients	153	26	
Age			0.061
<50	21 (13.73%)	2 (7.69%)	
50–59	60 (39.22%)	6 (23.08%)	
60–69	58 (37.91%)	12 (46.15%)	
70–79	11 (7.19%)	6 (23.08%)	
>80	3 (1.96%)	0 (0.00%)	
Gender			0.227
Male	127 (83.01%)	19 (73.08%)	
Female	26 (16.99%)	7 (26.92%)	
Tobacco use			0.116
No	52 (33.99%)	13 (50.00%)	
Yes	101 (66.01%)	13 (50.00%)	
Alcohol use			0.301
No	60 (39.22%)	13 (50.00%)	
Yes	93 (60.78%)	13 (50.00%)	
Arrhythmia			0.903
No	116 (75.82%)	20 (76.92%)	
Yes	37 (24.18%)	6 (23.08%)	
Pneumonia			0.367
No	139 (90.85%)	25 (96.15%)	
Yes	14 (9.15%)	1 (3.85%)	
Anastomotic leak			0.286
No	144 (94.12%)	23 (88.46%)	
Yes	9 (5.88%)	3 (11.54%)	
Adjuvant therapy			0.794
No	49 (32.03%)	9 (34.62%)	
Yes	104 (67.97%)	17 (65.38%)	
T stage			0.553
T1	10 (6.54%)	2 (7.69%)	
T2	21 (13.73%)	6 (23.08%)	
T3	97 (63.40%)	13 (50.00%)	
T4	25 (16.34%)	5 (19.23%)	
N stage			0.501
N0	70 (45.75%)	13 (50.00%)	
N1	53 (34.64%)	9 (34.62%)	
N2	18 (11.76%)	4 (15.38%)	
N3	12 (7.84%)	0 (0.00%)	
TNM stage			0.809
I	8 (5.23%)	2 (7.69%)	
II	67 (43.79%)	10 (38.46%)	
III	78 (50.98%)	14 (53.85%)	
Tumor location			0.142
Upper	19 (12.42%)	1 (3.85%)	
Middle	85 (55.56%)	12 (46.15%)	
Lower	49 (32.03%)	13 (50.00%)	
Tumor grade			0.066
Well	31 (20.26%)	1 (3.85%)	
Poorly	43 (28.10%)	6 (23.08%)	
Moderately	79 (51.63%)	19 (73.08%)	
Whether death			0.047^*^
No	67 (43.79%)	6 (23.08%)	
Yes	86 (56.21%)	20 (76.92%)	
OS	37.81 ± 23.01	27.10 ± 20.00	0.027^*^
Whether recurrence			0.071
No	88 (57.52%)	10 (38.46%)	
Yes	65 (42.48%)	16 (61.54%)	
RFS	30.58 ± 25.49	20.20 ± 21.06	0.051

* *p < 0.05*.

We also found that age more than 70 years, high N stage, poor grade, and advanced TNM stage were significantly associated with worse OS. However, there was no apparent influence of single components of MetS, such as BMI, hyperglycemia, hypertension, and TG, HDL-C, and LDL-C levels. The multivariate Cox analysis demonstrated that MetS was an independent factor (HR = 2.21; 95% CI: 1.27–3.86; *p* = 0.005). Meanwhile, age >70 years, poor grade, and upper tumor location were all identified as independent prognostic factors (Table 2).

**Table 2:** Association between metabolic syndrome (MetS) and overall survival (OS) in 179 patients in a univariate and multivariable analysis.

			**Univariable**			**Multivariable**	
**Variable**		**Hazard ratio**	**95% confidence interval**	***p***	**Hazard ratio**	**95% confidence interval**	***p***
Age	<50/50–59	0.91	0.46–1.78	0.776	0.99	1.48–2.06	0.979
	60–69/50–59	1.35	0.87–2.11	0.185	1.41	0.88–2.35	0.150
	70–79/50–59	1.91	1.01–3.61	0.047^*^	2.08	1.02–4.25	0.044^*^
	>80/50–59	5.39	1.64–17.69	0.005^**^	12.96	3.62–46.44	0.0001^***^
Gender	Female/male	1.28	0.80–2.05	0.306	–	–	–
Tobacco use	Yes/no	0.75	0.51–1.10	0.144	–	–	–
Alcohol use	Yes/no	0.86	0.59–1.27	0.457	–	–	–
Adjuvant therapy	Yes/no	1.93	1.22–3.04	0.004^**^	1.24	0.74–2.10	0.413
T stage	T1/T3	0.97	0.44–2.12	0.935	1.40	0.48–4.08	0.532
	T2/T3	1.05	0.60–1.82	0.870	1.26	0.65–2.45	0.501
	T4/T3	1.64	1.00–2.67	0.048^*^	1.69	0.93–3.08	0.083
N stage	N1/N0	2.04	1.31–3.18	0.001^***^	1.37	0.68–2.75	0.379
	N2/N0	2.05	1.14–3.70	0.017^*^	1.20	0.48–2.97	0.694
	N3/N0	2.97	1.42–6.19	0.003^**^	1.74	0.65–4.64	0.270
TNM stage	T1/T2	0.56	0.17–1.82	0.336	0.55	0.13–2.33	0.418
	T3/T2	2.03	1.35–3.06	0.0006^***^	1.68	0.77–3.68	0.191
Tumor location	Upper/middle	1.47	0.83–2.59	0.186	2.03	1.07–3.86	0.029^*^
	Lower/middle	0.88	0.57–1.35	0.561	0.66	0.41–1.07	0.088
Tumor grade	Well/moderately	0.99	0.57–1.70	0.961	1.41	0.75–2.66	0.287
	Poorly/moderately	1.63	1.06–2.50	0.024^*^	1.68	1.05–2.71	0.032^*^
MetS	With/without	1.73	1.06–2.82	0.027^*^	2.21	1.27–3.86	0.005^**^
BMI	Yes/no	1.30	0.86–1.96	0.220	–	–	–
Hyperglycemia	Yes/no	1.24	0.83–1.83	0.294	–	–	–
Hypertension	Yes/no	1.24	0.83–1.84	0.290	–	–	–
Triglycerides	Yes/no	1.11	0.65–1.89	0.701	–	–	–
HDL-C	Yes/no	0.95	0.59–1.54	0.848	–	–	–
LDL-C	Yes/no	1.07	0.72–1.59	0.752	–	–	–
Arrhythmia	Yes/no	1.12	0.73–1.72	0.608	–	–	–
Pneumonia	Yes/no	1.43	0.72–2.83	0.309	–	–	–
Anastomotic leak	Yes/no	1.30	0.60–2.80	0.503	–	–	–

* *p < 0.05*,

** *p < 0.01*,

*** *p < 0.001*.

We found that N stage, history of adjuvant therapy, hyperglycemia, T stage, and TNM stage were associated with RFS in univariate analysis, and MetS (HR =1.71; 95% CI: 0.99–2.96; *p* = 0.0554) influenced the RFS of patients. The multivariate Cox analysis demonstrated that adjuvant therapy (HR = 5.34; 95% CI: 2.23–12.77; *p* = 0.0002), TNM stage III (HR = 2.72; 95% CI: 1.12–6.61; *p* = 0.027), and MetS (HR = 2.39; 95% CI: 1.29–4.43; *p* = 0.005) were all significant risk factors (Table 3) for RFS.

**Table 3:** Association between metabolic syndrome (MetS) and recurrence-free survival (RFS) in 179 patients in a univariate and multivariable analysis.

			**Univariable**			**Multivariable**	
**Variable**		**Hazard ratio**	**95% confidence interval**	***p***	**Hazard ratio**	**95% confidence interval**	***p***
Age	<50/50–59	1.36	0.74–2.51	0.325	1.83	0.90–3.70	0.094
	60–69/50–59	0.75	0.45–1.26	0.283	0.83	0.46–1.48	0.525
	70–79/50–59	0.97	0.43–2.21	0.947	1.06	0.43–2.62	0.892
	>80/50–59	1.13	0.15–8.32	0.901	2.25	0.28–17.89	0.445
Gender	Female/male	0.75	0.41–1.39	0.359	–	–	–
Tobacco use	Yes/no	1.18	0.74–1.88	0.492	–	–	–
Alcohol use	Yes/no	1.31	0.83–2.06	0.247	–	–	–
Adjuvant therapy	Yes/no	8.50	3.69–19.55	0.0001^***^	5.34	2.23–12.77	0.0002^***^
T stage	T1/T3	1.13	0.48–2.65	0.776	1.18	0.36–3.82	0.787
	T2/T3	0.80	0.39–1.64	0.548	1.03	0.44–2.39	0.946
	T4/T3	2.38	1.41–4.04	0.001^**^	1.40	0.74–2.64	0.303
N stage	N1/N0	2.51	1.47–4.29	0.0001^***^	1.03	0.45–2.35	0.940
	N2/N0	3.26	1.67–6.39	0.0001^***^	1.11	0.41–2.99	0.833
	N3/N0	6.56	3.08–14.00	0.0001^***^	2.21	0.74–6.61	0.156
TNM stage	T1/T2	1.15	0.34–3.89	0.822	1.18	0.28–5.02	0.824
	T3/T2	3.67	2.18–6.18	0.0001^***^	2.72	1.12–6.61	0.027^*^
Tumor location	Upper/middle	1.50	0.79–2.83	0.217	1.37	0.65–2.86	0.406
	Lower/middle	0.85	0.52–1.39	0.513	0.58	0.34–1.00	0.051
Tumor grade	Well/moderately	0.76	0.39–1.46	0.403	1.02	0.47–2.21	0.959
	Poorly/moderately	1.00	0.60–1.66	0.989	0.86	0.49–1.51	0.597
MetS	With/without	1.71	0.99–2.96	0.055	2.39	1.29–4.43	0.005^**^
BMI	Yes/no	1.24	0.77–2.00	0.373	–	–	–
Hyperglycemia	Yes/no	1.58	1.02–2.46	0.040^*^	–	–	–
Hypertension	Yes/no	1.14	0.72–1.79	0.574	–	–	–
Triglycerides	Yes/no	1.04	0.57–1.93	0.888	–	–	–
HDL-C	Yes/no	0.68	0.38–1.24	0.211	–	–	–
LDL-C	Yes/no	0.93	0.59–1.48	0.759	–	–	–
Arrhythmia	Yes/no	0.99	0.59–1.66	0.981	–	–	–
Pneumonia	Yes/no	1.10	0.48–2.53	0.821	–	–	–
Anastomotic leak	Yes/no	1.03	0.42–2.54	0.952	–	–	–

* *p < 0.05*,

** *p < 0.01*,

*** *p < 0.001*.

### MetS May Have an Effect on the Tumor Microenvironment

**Figure 2:**
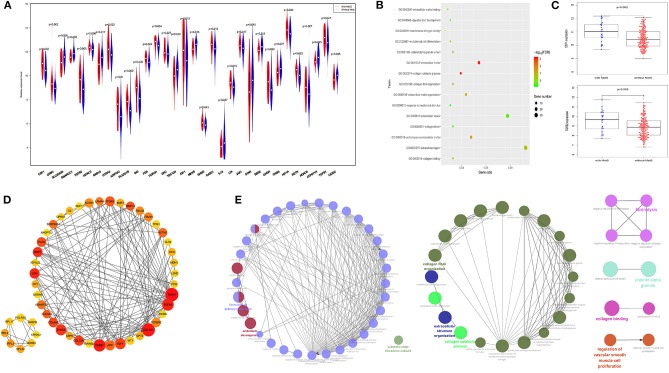
The extracellular matrix related pathways were significantly enriched. **(A)** The difference gene expression between the two groups. **(B)** The function enrichment analysis results of four module genes. **(C)** The expression of CSF1 and TGFβ2 were high in the MetS group. **(D)** The protein–protein interaction (PPI) for top 50 hub gene by cotoHubba. **(E)** The function enrichment analysis of the top 50 hub gene by ClueGO.

Mechanism-related gene sets of MetS were selected from MSigDB and analyzed within our groups (10, 33), and the results showed that differences in the expression of genes, such as IGF1, IGFALS, CSF1, TGFβ1, and TGFβ2, were associated with MetS (Figure 2A, Supplementary Table 1). Our module genes (MEgrey, MEsalmon, MEgreenyellow, and MEpurple) associated with MetS based on WGCNA were used to understand the molecular functions and pathways (Supplementary Figure 1). We found that extracellular matrix-related pathways were significantly enriched (Figure 2B). A PPI network of the top 50 hub genes and function enrichment were constructed and shown in Figures 2D,E. These results indicated that the effect of MetS on tumor prognosis might be associated with the tumor microenvironment.

**Figure 3:**
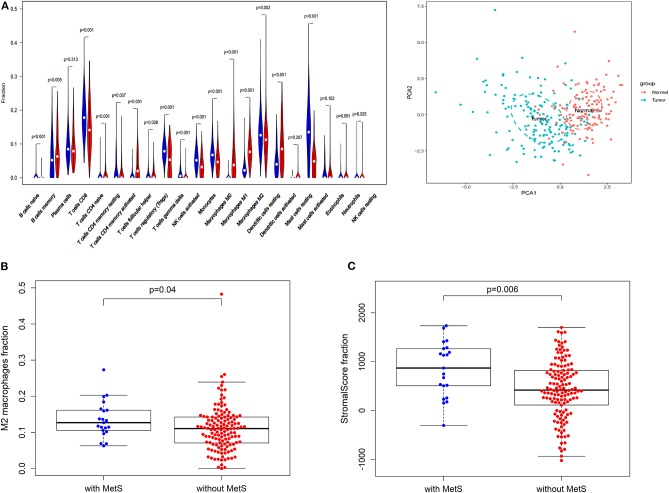
The effect of MetS on ESCC might be associated with tumor microenvironment. **(A)** The profiles of immune infiltration and principal component analysis (PCA) between tumor and normal tissues. **(B)** The MetS patients have a high infiltration of Macrophages M2. **(C)** The MetS patients have high stromal scores.

Immune infiltration and stromal scores can reflect the status of the tumor microenvironment. Based on CIBERSORT, unmatched samples with *p* > 0.05 were removed, and a total of 139 normal and 155 tumor samples were used for further analysis. Our results showed that there were significant differences in the abundance of various immune cells (Figure 3A). Principal component analysis (PCA) could better distinguish normal and tumor tissues (Figure 3A), indicating a good predictive value.

We found that only naive CD4 T cells and M0 macrophages were associated with low RFS (Supplementary Figure 2A). Next, we explored the correlation between clinical features, such as T stage (gamma delta T cells, *p* < 0.05), N stage (M1 macrophages and resting memory CD4 T cells, *p* < 0.05), TNM stage (activated memory CD4 T cells and gamma delta T cells, *p* < 0.05) and tumor grade (M0 macrophages, *p* < 0.05), and different immune cells (Supplementary Figure 2B). Above all, the MetS group had a high infiltration of M2 macrophages (*p* = 0.04, Figure 3B), which display alternatively activated states with pro-tumorigenic effects. Meanwhile, the MetS group was significantly associated with the stromal scores based on the ESTIMATE algorithm (*p* = 0.006, Figure 3C).

### Identification of a Six-lncRNA Signature

**Figure 4:**
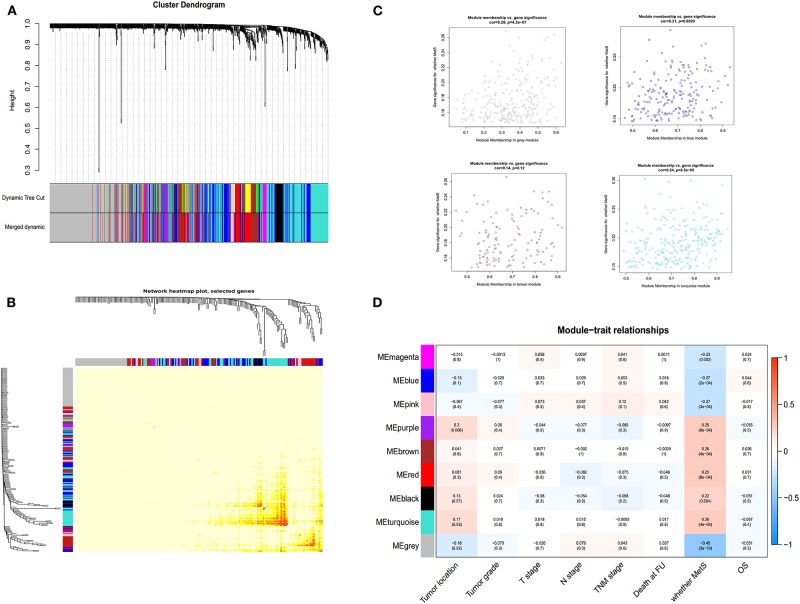
The selection of lncRNA module using WGCNA. The merged dynamic of lncRNA module **(A)**, network heatmap plot **(B)**, four-module membership with gene significant **(C)** and the module trait relationship **(D)**.

**Figure 5:**
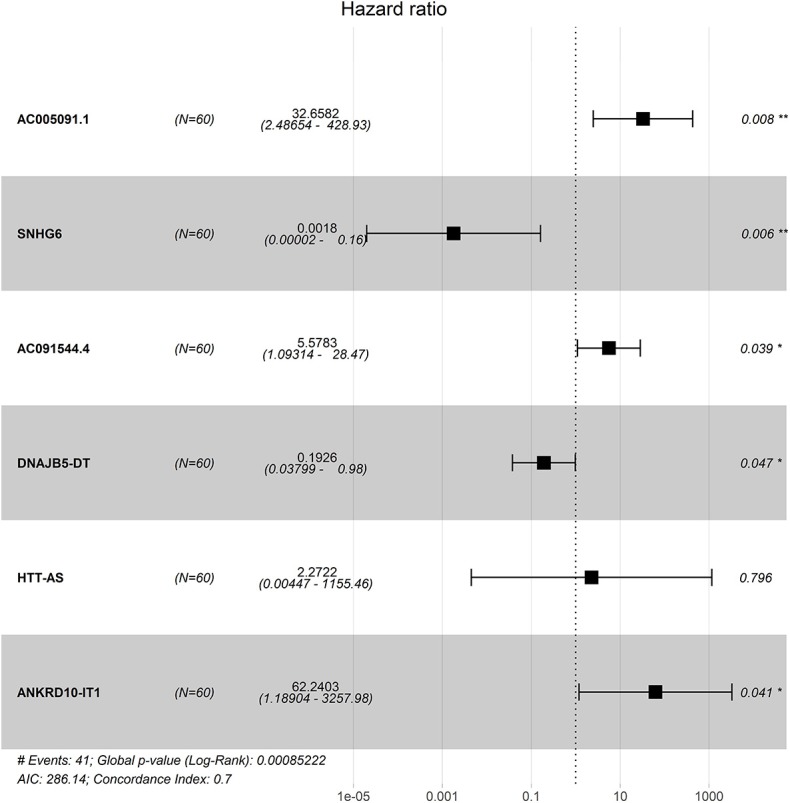
The forest plot for the six lncRNA signature.

**Figure 6:**
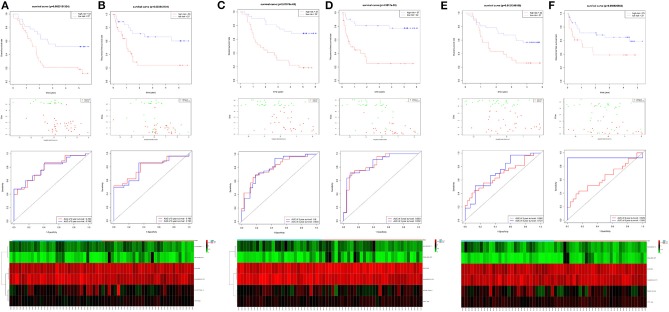
The identification and validation of the six-lncRNA signature. The OS and RFS, the distribution of patients' risk score, survival status, ROC curves, and heatmap of the six-lncRNA signature in the training cohort of 60 patients **(A,B)**, the validation cohort of 59 patients **(C,D)**, and 60 patients **(E,F)**.

Four hundred sixty-six downregulated and 623 upregulated lncRNAs were found (Supplementary Figures 3A,B). *z* scores were calculated in the MetS-related four-lncRNA module (MEgrey, MEblue, MEbrown, and MEturquoise) with univariate Cox PHR analysis (Figure 4). A total of 42 lncRNAs were selected with |*z*| scores > 2 and *p* < 0.05. Finally, a six-lncRNA signature was established by multivariable stepwise Cox PHR analysis (Figure 5). The six-lncRNA signature was used to calculate risk scores using estimated regression coefficients to divide the 60 ESCC patients into a high-risk group (*n* = 33) and a low-risk group (*n* = 27) with a cutoff point of 1.051. There was a significant difference between the high- and low-risk groups in terms of OS (*p* = 0.002, Figure 6A) and RFS (*p* = 0.003, Figure 6B). Time-dependent ROC curves and areas under ROC curves (AUCs) for the six-lncRNA signature were determined and showed an effective predictive value at 3- and 5-year OS (0.785 and 0.786, respectively, Figure 6A) and RFS (0.795 and 0.783, respectively, Figure 6B). Finally, the six-lncRNA signature (AC005091.1, SNHG6, AC091544.4, DNAJB5-DT, HTT-AS, and ANKRD10-IT1) with the best prognostic performance was selected and the estimated regression coefficients are as follows:

Risk score = (2.2544 × expression level of AC005091.1) + (−5.1919 × expression level of SNHG6) + (1.3410 × expression level of AC091544.4) + (−1.3675 × expression level of DNAJB5-DT) + (−4.0758 × expression level of HTT-AS) + (3.6637 × expression level of ANKRD10-IT1).

### Validation of the Six-lncRNA Signature

**Figure 7:**
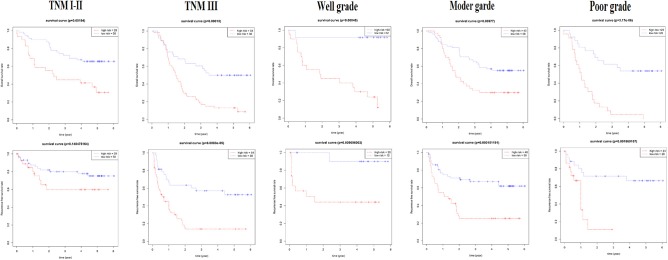
The subgroup of the six-lncRNA signature for 179 patients. The TNM stage and tumor grade were used to analyze the predictive ability for the six-lncRNA signature.

For verification, we found that the six-lncRNA signature could effectively distinguish the high- and low-risk groups in 59 patients from GSE53624 (OS: *p* = 2.87E−05; RFS: *p* = 4.38E−05, Figures 6C,D) and 60 patients from GSE53622 (OS: *p* = 0.012; RFS: *p* = 0.05, Figures 6E,F). The 80 ESCC patients from TCGA (OS: *p* = 0.03; RFS: *p* = 0.05, Supplementary Figure 4A), the 179 ESCC patients (OS: *p* = 6.9E−09; RFS: *p* = 1.37e-07, Supplementary Figure 4B), and randomly grouping all showed an effective predictive ability (Supplementary Figures 4C,D). In addition, we investigated the predictive power in subgroups based on TNM stage and tumor grade and identified a moderate performance for risk predicting (Figure 7).

### The Six-lncRNA Signature Is an Independent Risk Factor

Multivariate Cox analysis demonstrated that the six-lncRNA signature could be regarded as an independent predictive factor for OS in the training and validation cohort (Table 4 and Supplementary Tables 2–4). A total of 179 patients demonstrated that the six-lncRNA signature could be regarded as an independent predictive factor for both OS (HR = 3.97; 95% CI: 2.47–6.36; *p* = 0.0001) and RFS (HR = 3.23; 95% CI: 1.95–5.35; *p* = 0.0001), as could be MetS for OS (HR = 2.67; 95% CI: 1.48–4.83; *p* = 0.001) and RFS (HR = 2.40; 95% CI: 1.25–4.60; *p* = 0.008, Table 4 and Supplementary Table 5). A total of 80 patients from TCGA demonstrated that the six-lncRNA signature was also an independent prognostic factor for OS (HR = 5.37; 95% CI: 1.02–28.41; *p* = 0.047) and RFS (HR = 3.47; 95% CI: 1.04–11.62; *p* = 0.043, Table 4 and Supplementary Table 6).

**Table 4:** The multivariable analysis for MetS and six-lncRNA signature.

**Group**	**Factors**	**Overall survival (OS)**			**Recurrence-free survival (RFS)**		
		**HR**	**95% CI**	***p***	**HR**	**95% CI**	***p***
60 patients(training)	MetS	4.26	1.13–16.03	0.031^*^	5.87	1.20–28.62	0.028^*^
	Six-lncRNA signature	3.49	1.57–7.77	0.002^**^	5.27	1.56–17.75	0.007^**^
59 patients (validation 1)	MetS	9.82	1.96–49.10	0.005^**^	84.26	5.67–1253.30	0.001^**^
	Six-lncRNA signature	7.21	2.02–25.76	0.002^**^	104.03	7.90–1370.66	0.0004^***^
60 patients (validation 2)	MetS	2.38	0.75–7.54	0.141	1.32	0.42–4.16	0.635
	Six-lncRNA signature	3.00	1.18–7.62	0.02^*^	3.37	0.96–11.81	0.058
Total 179 patients	MetS	2.67	1.48–4.83	0.001^**^	2.40	1.25–4.60	0.008^**^
	Six-lncRNA signature	3.97	2.47–6.36	0.0001^***^	3.23	1.95–5.35	0.0001^***^
80 patients from TCGA	Six-lncRNA signature	5.37	1.02–28.41	0.047^*^	3.47	1.04–11.62	0.043^*^

* *p < 0.05*,

** *p < 0.01*,

*** *p < 0.001*;

*HR, hazard ratio; 95% CI, 95% confidence interval*.

**Figure 8:**
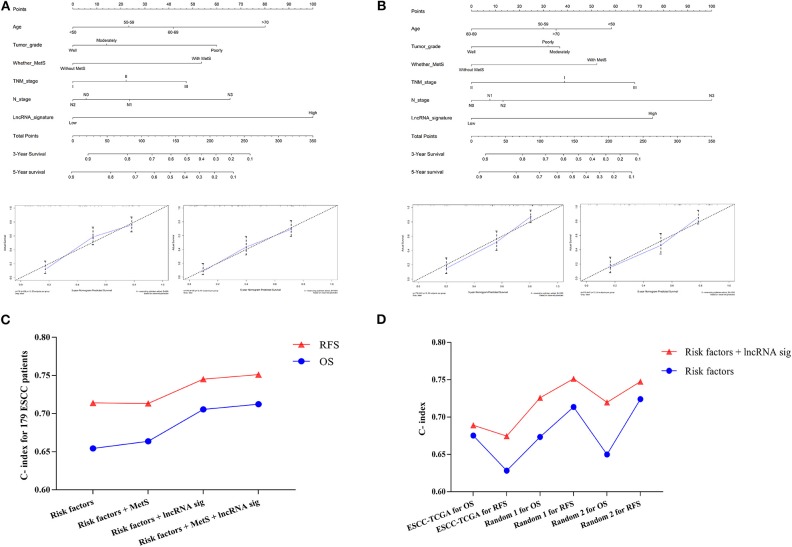
The establishment of the clinical predictive model. The prognostic nomogram was established by Age, Tumor grade, MetS, TNM stage, N stage, and lncRNA signature for OS **(A)** and RFS **(B)**; the calibration curves for the nomogram for 3 and 5 years were also shown. The C-indexes were calculated and indicated that the six-lncRNA signature could enhance the predictive power in both training **(C)** and validation group **(D)**.

Next, we selected MetS, lncRNA signature, age, tumor grade, TNM stage, and N stage to establish a prognostic nomogram to predict 3- and 5-year OS and RFS in 179 ESCC patients (Figures 8A,B). The C-index was 0.71 for OS and 0.75 for RFS (Figure 8C). Calibration curves for the nomogram for 3- and 5-year survival showed good agreement between the actual observation and prediction (Figures 8A,B). The lncRNA signature and MetS could all enhance the predictive power. Finally, the random group was used for internal validation. The ESCC patients with available clinical data from TCGA were classified into an external validation group. These results all showed a moderate predictive value (Figure 8D).

## Discussion

The prevalence of excess body weight and associated cancer burden have been increased over the past several decades globally (34). Nearly 30% of esophageal adenocarcinoma cases are attributable to excess body weight (body mass index, BMI ≥25 kg/m^2^) in 2012 worldwide (35). Obesity is considered an indicator of MetS and is associated with a high risk of esophageal carcinoma (16, 17). China has the highest incidence of esophageal cancer, especially ESCC, and the most important risk factors may be inadequate intake of fruits and vegetables, poor nutritional status, and drinking hot beverages and food (36, 37). Currently, based on population analysis, some studies (13, 15, 18) have indicated that MetS is associated with the prognosis of ESCC patients.

The effect of MetS on prognosis was largely mediated by single metabolic components such as hyperglycemia and dyslipidemia (13). Our study showed similar results but mainly reflected in the overall effect, not the single factor. The reason might be associated with the number and characteristics of the population.

**Figure 9:**
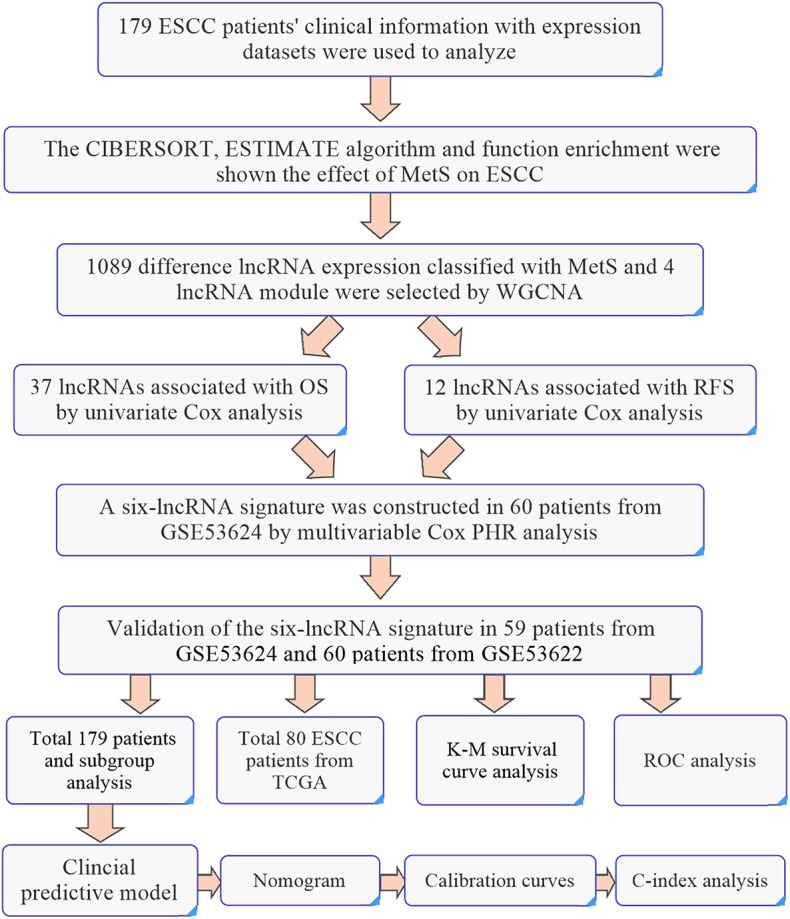
The workflow of identification of six-lncRNA signature.

These 179 ESCC patients with expression profiles allow us to explore the possible mechanisms from a molecular perspective and the detailed workflow is shown in Figure 9. The biological alterations associated with MetS that influence cancer development and the changes of related gene expression through high glucose, insulin resistance, abnormal cytokines or hormonal level, chronic inflammation, and oxidative stress (9, 10). Insulin resistance-associated factors, such as IGF1 and IGFALS, and inflammation-related factors, such as CSF1 and TGFβ2, were all highly enriched in the MetS group (Figures 2A,C). We know that the high expression of IGF1 and inflammatory factors can promote tumor development by changing the tumor microenvironment (10, 38). The cytokines CSF1 and TGFβ have the ability to promote the transformation of M2 macrophages (39, 40). Immune infiltration analysis has also shown that the M2 macrophages were higher in the MetS group. The macrophages can be divided into three subtypes (M0, M1, and M2), where M0 is the unactivated subtype and can be differentiated into two M1 and M2 activated subtypes. M1 macrophages are pro-inflammatory and participate in the host innate immunity to kill tumor cells (41, 42). M2 macrophages are regarded as tumor-promotive and are correlated with poor prognosis in various cancers, including ESCC (43, 44). M2 macrophages could promote tumor metastasis by inducing epithelial–mesenchymal transition (EMT) or secreting cytokines such as IL-1β (45). The infiltrated immune cell can induce the host immune response to inhibit or promote the progression of tumor cells in the tumor microenvironment (42). Hence, the high infiltration rate of M2 macrophages in the MetS group might be associated with tumor microenvironment. Furthermore, the enrichment of ECM-related pathway and high stromal scores were also all found in the MetS group (Figures 2B,E, 3C). The extracellular matrix (ECM) regulated tissue development and homeostasis, and its dysregulation contributed to tumor progression (46). The ECM remodeling with secretion of fibronectin, collagens, and deposition of matrix proteins had an effect on the microenvironment during tumor progression (47–49). Above all, the change of tumor microenvironment in the MetS group might be the possible mechanism on tumor progression and a reason for the poor prognosis.

The recent studies have shown that immune cell infiltration could be used to establish a prognosis model in prostate cancer and colorectal cancer (42, 50). Hence, we also tried to establish a prognosis predictive signature based on immune infiltration profiles. Immune cell signature was established but with low predictive power (Supplementary Figures 5, 6). Then, we constructed a lncRNA signature and found that the six-lncRNA signature had an available predictive ability for prognosis. The six-lncRNA signature was established with forward stepwise approach based on the 42 lncRNAs. Starting with the lncRNA “ANKRD10-IT1” with the largest univariate *z* score, we gradually added lncRNA and evaluated the prognostic performance. The process was repeated until no improvement can be found. Among the six-lncRNA signature components, SNHG6 has an effect on tumor progression in various cancer types, such as colorectal cancer (51), breast cancer (52), lung cancer (53), and ESCC (54). ANKRD10-IT1 was also regarded as a potential prognostic biomarker in hepatocellular carcinoma (55). The lncRNA signature and MetS were all associated with prognosis and could also serve as risk factor with available predictive ability (Figure 8). Next, we found that the combination use of immune infiltration signature only slightly enhances the predictive efficiency on prognosis (Supplementary Figure 5). These results indicated that the lncRNA signature might be more suitable for ESCC, but the immune infiltration signature still needs to be further explored.

The lncRNAs have an effect on epigenetic regulation including transcription, post-transcriptional regulation, and post-translational modification of proteins. They are also regarded as biomarkers for tumors and therapeutic targets (56). A number of esophageal cancer-related lncRNAs were dysregulated in obesity such as ANRIL, H19, and HOTAIR, suggesting that obesity-associated lncRNAs may promote development of cancer (57–59). Obesity is one of the components of MetS and is mainly associated with the occurrence and development of esophageal adenocarcinoma (33). Although the pathogenesis of ESCC is different from that of esophageal adenocarcinoma, the progression may have a similar mechanism under the influence of obesity and MetS because of the change of tumor microenvironment. Hence, the MetS and related lncRNA signature might be appropriate to reflect the development of ESCC and have available predictive power. In total, our results indicated that MetS and multifunction lncRNA could be involved in the development of ESCC.

The previous studies all had the epidemiological perspective of exploring the relationship between MetS and ESCC (13, 15, 18). Our study is the first to explore the correlation from points of epidemiology and molecular mechanism and to successfully construct a six-lncRNA signature with a significant clinical application value. However, there are several limitations to our study. First, preoperative disease control status, pathogenic factors, and possible selection bias among patients with MetS might have influenced the results. Second, the difference in MetS diagnostic criteria and the limitations in the available clinical information for ESCC patients in the public database restricted further research.

## Conclusions

Our results indicated that MetS was associated with poor prognosis in ESCC patients, and the possible mechanism was related to changes in the tumor microenvironment. MetS and the six-lncRNA signature could also serve as independent risk factors with clinical application value.

## Data Availability Statement

The data that support the findings of this study come from the public free-charged database, and the new clinical information will be available from the corresponding author, without undue reservation, to any qualified researcher.

## Ethics Statement

All samples were collected with signed informed consent and were approved by Medical ethics committee of the Cancer institute and Hospital, Chinese Academy of Medical Sciences. The patients/participants provided their written informed consent to participate in this study.

## Author Contributions

YL, CL, and JH: study design. YL, LW, and HL: data collections. YL: data analysis. YL, CL, and JH: writing. All authors reviewed the manuscript.

### Conflict of Interest

The authors declare that the research was conducted in the absence of any commercial or financial relationships that could be construed as a potential conflict of interest.
