# Supplementary material for: The Prognostic Significance of Metabolic Syndrome and a Related Six-lncRNA Signature in Esophageal Squamous Cell Carcinoma
Source: Front Oncol. 2020 Feb 18;10:61. doi: 10.3389/fonc.2020.00061 (PMC7040247; doi:10.3389/fonc.2020.00061)
Supplement: Supplementary file 1 [file Table_1.DOCX]

Supplement table 1. The mechanism-associated gene sets between MetS and tumor

WANG_CLASSIC_ADIPOGENIC_TARGETS_OF_PPARG

GO_RESPONSE_TO_LEPTIN

GO_CELLULAR_RESPONSE_TO_LEPTIN_STIMULUS

GO_CELLULAR_RESPONSE_TO_INSULIN_STIMULUS

GO_INSULIN_LIKE_GROWTH_FACTOR_BINDING

BIOCARTA_IL6_PATHWAY

BIOCARTA_INFLAM_PATHWAY
