# Supplementary material for: The Prognostic Significance of Metabolic Syndrome and a Related Six-lncRNA Signature in Esophageal Squamous Cell Carcinoma
Source: Front Oncol. 2020 Feb 18;10:61. doi: 10.3389/fonc.2020.00061 (PMC7040247; doi:10.3389/fonc.2020.00061)
Supplement: Supplementary file 2 [file Table_2.DOCX]

Supplement Table 2 Association between six-lncRNA signature and overall survival(OS) in training cohort (60 patients) in a univariate and multivariable analysis

|  |  |  | Univariable |  |  | Multivariable |  |
| --- | --- | --- | --- | --- | --- | --- | --- |
| Variable |  | Hazard ratio | 95% confidence interval | *p* | Hazard ratio | 95% confidence interval | *p* |
| Age | <50/50-59 | 1.23 | 0.43-3.55 | 0.697 | 1.81 | 0.47-7.01 | 0.389 |
|  | 60-69/50-59 | 1.45 | 0.68-3.10 | 0.335 | 2.62 | 1.00-6.89 | 0.050 |
|  | 70-79/50-59 | 2.23 | 0.89-5.54 | 0.085 | 4.3 | 1.30-14.18 | 0.016* |
| Gender | Female/male | 1.21 | 0.53-2.73 | 0.653 | ­— | — | — |
| Tobacco use | Yes/no | 0.75 | 0.40-1.41 | 0.370 | — | — | — |
| Alcohol use | Yes/no | 0.9 | 0.47-1.70 | 0.739 | — | — | — |
| Adjuvant therapy | Yes/no | 1.88 | 0.90-3.95 | 0.094 | 1.64 | 0.68-3.94 | 0.270 |
| T stage | T1/T3 | 1.09 | 0.36-3.26 | 0.883 | 0.51 | 0.08-3.41 | 0.486 |
|  | T2/T3 | 2.61 | 1.18-5.76 | 0.018* | 1.63 | 0.43-6.15 | 0.467 |
|  | T4/T3 | 3.02 | 1.38-6.60 | 0.005** | 0.92 | 0.34-2.51 | 0.867 |
| N stage | N1/N0 | 2.33 | 1.13-4.80 | 0.021* | 1.53 | 0.33-7.05 | 0.583 |
|  | N2/N0 | 2.23 | 0.79-6.29 | 0.129 | 0.68 | 0.07-6.87 | 0.747 |
|  | N3/N0 | 2.9 | 0.93-9.01 | 0.065 | 4.2 | 0.47-37.83 | 0.200 |
| TNM stage | T1/T2 | 1.55 | 0.35-6.89 | 0.563 | 1.49 | 0.14-15.88 | 0.743 |
|  | T3/T2 | 2.89 | 1.46-5.69 | 0.002** | 2.52 | 0.51-12.49 | 0.257 |
| Tumor location | Upper/middle | 1.09 | 0.42-2.89 | 0.855 | 1.44 | 0.40-5.12 | 0.575 |
|  | lower/middle | 1.45 | 0.74-2.87 | 0.282 | 1.31 | 0.54-3.18 | 0.548 |
| Tumor grade | Well/moderately | 1.13 | 0.48-2.67 | 0.785 | 0.49 | 0.16-1.50 | 0.209 |
|  | Poorly/moderately | 1.76 | 0.88-3.53 | 0.109 | 2.35 | 0.91-6.06 | 0.078 |
| MetS | With/without | 2.24 | 0.98-5.11 | 0.054 | 4.26 | 1.13-16.03 | 0.031* |
| LncRNA-signature | High/low | 2.71 | 1.39-5.28 | 0.003** | 3.49 | 1.57-7.77 | 0.002** |
| BMI | Yes/no | 1.72 | 0.91-3.27 | 0.094 | — | — | — |
| Hyperglycemia | Yes/no | 1.68 | 0.88-3.18 | 0.112 | — | — | — |
| Hypertension | Yes/no | 1.01 | 0.54-1.88 | 0.980 | — | — | — |
| Triglycerides | Yes/no | 1.67 | 0.70-4.00 | 0.245 | — | — | — |
| HDL-C | Yes/no | 0.75 | 0.35-1.64 | 0.476 | — | — | — |
| LDL-C | Yes/no | 1.39 | 0.75-2.60 | 0.300 | — | — | — |
| Arrhythmia | Yes/no | 1.27 | 0.63-2.54 | 0.500 | — | — | — |
| Pneumonia | Yes/no | 3.6 | 1.57-8.24 | 0.002** | — | — | — |
| Anastomotic leak | Yes/no | 2.23 | 0.87-5.73 | 0.095 | — | — | — |

**p*<0.05, ***p*<0.01, ****p*<0.001

Association between six-lncRNA signature and recurrence free survival (RFS) in training cohort (60 patients) in a univariate and multivariable analysis

|  |  |  | Univariable |  |  | Multivariable |  |
| --- | --- | --- | --- | --- | --- | --- | --- |
| Variable |  | Hazard ratio | 95% confidence interval | *p* | Hazard ratio | 95% confidence interval | *p* |
| Age | <50/50-59 | 1.34 | 0.46-3.85 | 0.591 | 3.25 | 0.68-15.45 | 0.137 |
|  | 60-69/50-59 | 0.73 | 0.30-1.75 | 0.477 | 5.03 | 1.27-20.03 | 0.021* |
|  | 70-79/50-59 | 1.25 | 0.43-3.62 | 0.676 | 5.84 | 0.94-36.49 | 0.058 |
| Gender | Female/male | 1.02 | 0.39-2.66 | 0.973 | ­— | — | — |
| Tobacco use | Yes/no | 0.59 | 0.28-1.21 | 0.151 | — | — | — |
| Alcohol use | Yes/no | 0.94 | 0.44-1.97 | 0.859 | — | — | — |
| Adjuvant therapy | Yes/no | 6.32 | 1.90-21.00 | 0.002** | 9.44 | 2.11-42.29 | 0.003** |
| T stage | T1/T3 | 1.82 | 0.58-5.71 | 0.306 | 0.71 | 0.07-6.64 | 0.760 |
|  | T2/T3 | 2.20 | 0.76-6.37 | 0.145 | 1.05 | 0.20-5.41 | 0.952 |
|  | T4/T3 | 5.25 | 2.17-12.70 | 0.0002*** | 2.61 | 0.68-10.10 | 0.163 |
| N stage | N1/N0 | 3.36 | 1.39-8.17 | 0.007** | 2.80 | 0.38-20.48 | 0.311 |
|  | N2/N0 | 3.66 | 1.09-12.35 | 0.036* | 6.07 | 0.39-94.87 | 0.198 |
|  | N3/N0 | 5.91 | 1.74-20.15 | 0.004** | 12.24 | 0.56-268.80 | 0.112 |
| TNM stage | T1/T2 | 3.49 | 0.72-16.84 | 0.119 | 4.22 | 0.24-73.72 | 0.324 |
|  | T3/T2 | 5.29 | 2.19-12.74 | 0.0002*** | 1.20 | 0.13-11.38 | 0.871 |
| Tumor location | Upper/middle | 1.42 | 0.52-3.84 | 0.494 | 1.75 | 0.46-6.75 | 0.413 |
|  | lower/middle | 1.30 | 0.56-3.02 | 0.540 | 1.16 | 0.40-3.40 | 0.784 |
| Tumor grade | Well/moderately | 1.68 | 0.61-4.61 | 0.311 | 1.54 | 0.40-5.95 | 0.534 |
|  | Poorly/moderately | 1.91 | 0.84-4.37 | 0.123 | 3.38 | 0.93-12.23 | 0.063 |
| MetS | With/without | 1.65 | 0.63-4.34 | 0.308 | 5.87 | 1.20-28.62 | 0.028* |
| LncRNA-signature | High/low | 3.03 | 1.37-6.67 | 0.006** | 5.27 | 1.56-17.75 | 0.007** |
| BMI | Yes/no | 1.04 | 0.48-2.28 | 0.916 | — | — | — |
| Hyperglycemia | Yes/no | 1.70 | 0.82-3.54 | 0.157 | — | — | — |
| Hypertension | Yes/no | 0.96 | 0.47-1.99 | 0.922 | — | — | — |
| Triglycerides | Yes/no | 1.26 | 0.44-3.62 | 0.669 | — | — | — |
| HDL-C | Yes/no | 0.62 | 0.24-1.63 | 0.337 | — | — | — |
| LDL-C | Yes/no | 1.36 | 0.65-2.82 | 0.411 | — | — | — |
| Arrhythmia | Yes/no | 1.50 | 0.67-3.37 | 0.327 | — | — | — |
| Pneumonia | Yes/no | 1.74 | 0.52-5.85 | 0.367 | — | — | — |
| Anastomotic leak | Yes/no | 1.56 | 0.47-5.19 | 0.469 | — | — | — |

**p*<0.05, ***p*<0.01, ****p*<0.001
