# Supplementary material for: The Prognostic Significance of Metabolic Syndrome and a Related Six-lncRNA Signature in Esophageal Squamous Cell Carcinoma
Source: Front Oncol. 2020 Feb 18;10:61. doi: 10.3389/fonc.2020.00061 (PMC7040247; doi:10.3389/fonc.2020.00061)
Supplement: Supplementary file 3 [file Table_3.DOCX]

Supplement Table 3 Association between six-lncRNA signature and overall survival(OS) in validation cohort (59 patients) in a univariate and multivariable analysis

|  |  |  | Univariable |  |  | Multivariable |  |
| --- | --- | --- | --- | --- | --- | --- | --- |
| Variable |  | Hazard ratio | 95% confidence interval | *p* | Hazard ratio | 95% confidence interval | *p* |
| Age | <50/50-59 | 1.21 | 0.40-3.69 | 0.733 | 0.38 | 0.07-2.04 | 0.259 |
|  | 60-69/50-59 | 1.02 | 0.46-2.25 | 0.960 | 0.63 | 0.12-3.23 | 0.579 |
|  | 70-79/50-59 | 2.3 | 0.52-10.18 | 0.272 | 0.52 | 0.03-9.80 | 0.662 |
| Gender | Female/male | 1.29 | 0.58-2.88 | 0.529 | ­— | — | — |
| Tobacco use | Yes/no | 1.05 | 0.50-2.23 | 0.891 | — | — | — |
| Alcohol use | Yes/no | 1.18 | 0.58-2.40 | 0.641 | — | — | — |
| Adjuvant therapy | Yes/no | 1.66 | 0.72-3.84 | 0.236 | 2.65 | 0.61-11.62 | 0.195 |
| T stage | T1/T3 | 2.84 | 0.64-12.55 | 0.169 | 6.57 | 0.34-125.32 | 0.211 |
|  | T2/T3 | 0.3 | 0.07-1.30 | 0.108 | 0.19 | 0.02-2.33 | 0.193 |
|  | T4/T3 | 1.2 | 0.55-2.59 | 0.651 | 0.69 | 0.14-3.46 | 0.655 |
| N stage | N1/N0 | 1.85 | 0.83-4.14 | 0.134 | 1.50 | 0.29-7.86 | 0.632 |
|  | N2/N0 | 1.35 | 0.43-4.25 | 0.604 | 1.25 | 0.22-7.04 | 0.803 |
|  | N3/N0 | 4.13 | 1.30-13.13 | 0.016* | 3.73 | 0.36-38.41 | 0.268 |
| TNM stage | T1/T2 | 0.76 | 0.10-6.10 | 0.798 | 1.05 | 0.03-31.84 | 0.977 |
|  | T3/T2 | 1.94 | 0.86-4.34 | 0.108 | 0.72 | 0.11-4.85 | 0.735 |
| Tumor location | Upper/middle | 2.36 | 0.92-6.01 | 0.072 | 2.87 | 0.80-10.24 | 0.104 |
|  | lower/middle | 0.91 | 0.40-2.03 | 0.809 | 0.54 | 0.16-1.83 | 0.322 |
| Tumor grade | Well/moderately | 1.25 | 0.51-3.07 | 0.624 | 2.12 | 0.51-8.88 | 0.303 |
|  | Poorly/moderately | 1.5 | 0.67-3.35 | 0.319 | 2.85 | 0.77-10.61 | 0.118 |
| MetS | With/without | 1.28 | 0.45-3.65 | 0.649 | 9.82 | 1.96-49.10 | 0.0054** |
| LncRNA-signature | High/low | 4.43 | 2.07-9.45 | 0.0001*** | 7.21 | 2.02-25.76 | 0.0024** |
| BMI | Yes/no | 1.42 | 0.64-3.18 | 0.387 | — | — | — |
| Hyperglycemia | Yes/no | 0.83 | 0.41-1.71 | 0.620 | — | — | — |
| Hypertension | Yes/no | 0.8 | 0.36-1.79 | 0.593 | — | — | — |
| Triglycerides | Yes/no | 1.18 | 0.48-2.87 | 0.719 | — | — | — |
| HDL-C | Yes/no | 0.00 | 0.00-Inf | 0.997 | — | — | — |
| LDL-C | Yes/no | 0.85 | 0.39-1.84 | 0.680 | — | — | — |
| Arrhythmia | Yes/no | 1.48 | 0.69-3.21 | 0.315 | — | — | — |
| Pneumonia | Yes/no | 0.41 | 0.06-2.98 | 0.376 | — | — | — |
| Anastomotic leak | Yes/no | 0.84 | 0.20-3.50 | 0.805 | — | — | — |

**p*<0.05, ***p*<0.01, ****p*<0.001

Association between six-lncRNA signature and recurrence free survival (RFS) in validation cohort (59 patients) in a univariate and multivariable analysis

|  |  |  | Univariable |  |  | Multivariable |  |
| --- | --- | --- | --- | --- | --- | --- | --- |
| Variable |  | Hazard ratio | 95% confidence interval | *p* | Hazard ratio | 95% confidence interval | *p* |
| Age | <50/50-59 | 1.22 | 0.39-3.79 | 0.731 | 0.04 | 0.00-0.86 | 0.039* |
|  | 60-69/50-59 | 0.86 | 0.36-2.05 | 0.735 | 0.08 | 0.01-0.58 | 0.012* |
|  | 70-79/50-59 | 0 | 0.00-Inf 0 | 0.997 | 0.00 | 0.00-Inf | 0.998 |
| Gender | Female/male | 0.72 | 0.25-2.11 | 0.554 | ­— | — | — |
| Tobacco use | Yes/no | 1.91 | 0.72-5.11 | 0.194 | — | — | — |
| Alcohol use | Yes/no | 1.4 | 0.62-3.18 | 0.415 | — | — | — |
| Adjuvant therapy | Yes/no | 6.28 | 1.48-26.68 | 0.012* | 8.06 | 0.76-85.61 | 0.083 |
| T stage | T1/T3 | 1.37 | 0.18-10.52 | 0.764 | 3786052.4 | 0.00-Inf | 0.998 |
|  | T2/T3 | 0.22 | 0.03-1.64 | 0.138 | 0.01 | 0.00-1.55 | 0.072 |
|  | T4/T3 | 1.8 | 0.78-4.18 | 0.171 | 0.2 | 0.03-1.55 | 0.123 |
| N stage | N1/N0 | 1.57 | 0.61-4.08 | 0.352 | 2.51 | 0.22-29.07 | 0.460 |
|  | N2/N0 | 2.08 | 0.63-6.93 | 0.232 | 2.95 | 0.32-27.32 | 0.340 |
|  | N3/N0 | 6.08 | 1.79-20.65 | 0.003** | 59.14 | 3.63-963.33 | 0.004** |
| TNM stage | T1/T2 | 2.19 | 0.23-21.09 | 0.496 | 0 | 0.00-Inf 0 | 0.999 |
|  | T3/T2 | 5.19 | 1.54-17.46 | 0.007** | 2.6 | 0.17-38.93 | 0.488 |
| Tumor location | Upper/middle | 1.84 | 0.67-5.04 | 0.233 | 0.82 | 0.18-3.79 | 0.799 |
|  | lower/middle | 0.35 | 0.12-1.06 | 0.063 | 0.02 | 0.00-0.27 | 0.002** |
| Tumor grade | Well/moderately | 0.71 | 0.24-2.15 | 0.550 | 4.05 | 0.55-29.67 | 0.168 |
|  | Poorly/moderately | 0.76 | 0.29-1.96 | 0.570 | 1.76 | 0.30-10.40 | 0.532 |
| MetS | With/without | 1.74 | 0.60-5.07 | 0.311 | 84.26 | 5.67-1253.30 | 0.001** |
| LncRNA-signature | High/low | 5.27 | 2.17-12.82 | 0.0002*** | 104.03 | 7.90-1370.66 | 0.0004*** |
| BMI | Yes/no | 1.34 | 0.53-3.35 | 0.535 | — | — | — |
| Hyperglycemia | Yes/no | 1.61 | 0.74-3.54 | 0.232 | — | — | — |
| Hypertension | Yes/no | 0.57 | 0.21-1.52 | 0.260 | — | — | — |
| Triglycerides | Yes/no | 1.81 | 0.72-4.55 | 0.203 | — | — | — |
| HDL-C | Yes/no | 0.4 | 0.05-2.98 | 0.372 | — | — | — |
| LDL-C | Yes/no | 0.52 | 0.20-1.39 | 0.194 | — | — | — |
| Arrhythmia | Yes/no | 1.63 | 0.68-3.91 | 0.273 | — | — | — |
| Pneumonia | Yes/no | 0.51 | 0.07-3.79 | 0.511 | — | — | — |
| Anastomotic leak | Yes/no | 1.04 | 0.24-4.41 | 0.959 | — | — | — |

**p*<0.05, ***p*<0.01, ****p*<0.001
