# Supplementary material for: The Prognostic Significance of Metabolic Syndrome and a Related Six-lncRNA Signature in Esophageal Squamous Cell Carcinoma
Source: Front Oncol. 2020 Feb 18;10:61. doi: 10.3389/fonc.2020.00061 (PMC7040247; doi:10.3389/fonc.2020.00061)
Supplement: Supplementary file 4 [file Table_4.DOCX]

Supplement Table 4 Association between six-lncRNA signature and overall survival(OS) in validation cohort (60 patients) in a univariate and multivariable analysis

|  |  |  | Univariable |  |  | Multivariable |  |
| --- | --- | --- | --- | --- | --- | --- | --- |
| Variable |  | Hazard ratio | 95% confidence interval | *p* | Hazard ratio | 95% confidence interval | *p* |
| Age | <50/50-59 | 0.44 | 0.10-2.00 | 0.286 | 0.51 | 0.09-2.87 | 0.441 |
|  | 60-69/50-59 | 1.55 | 0.70-3.41 | 0.280 | 1.93 | 0.59-6.31 | 0.274 |
|  | 70-79/50-59 | 1.11 | 0.30-4.02 | 0.877 | 0.99 | 0.16-6.01 | 0.992 |
|  | >80/50-59 | 4.14 | 0.88-19.41 | 0.071 | 10.2 | 6 1.20-87.98 | 0.033* |
| Gender | Female/male | 1.41 | 0.61-3.24 | 0.424 | ­— | — | — |
| Tobacco use | Yes/no | 0.56 | 0.28-1.12 | 0.100 | — | — | — |
| Alcohol use | Yes/no | 0.58 | 0.29-1.15 | 0.117 | — | — | — |
| Adjuvant therapy | Yes/no | 2.14 | 0.96-4.76 | 0.062 | 2.07 | 0.54-7.99 | 0.291 |
| T stage | T1/T3 | 0.36 | 0.05-2.65 | 0.316 | 5.65 | 0.17-188.67 | 0.333 |
|  | T2/T3 | 0.82 | 0.29-2.34 | 0.705 | 1.01 | 0.18-5.73 | 0.992 |
|  | T4/T3 | 1.3 | 0.18-9.60 | 0.796 | 0.64 | 0.06-7.10 | 0.718 |
| N stage | N1/N0 | 1.87 | 0.85-4.10 | 0.120 | 0.84 | 0.06-11.66 | 0.897 |
|  | N2/N0 | 2.52 | 0.99-6.44 | 0.052 | 0.60 | 0.02-16.07 | 0.761 |
|  | N3/N0 | 1.52 | 0.20-11.74 | 0.685 | 2.01 | 0.05-75.82 | 0.705 |
| TNM stage | T1/T3 | 0.00 | 0.00-Inf 0 | 0.997 | 0.00 | 0.00-Inf | 9968 |
|  | T2/T3 | 1.71 | 0.86-3.40 | 0.126 | 2.23 | 0.14-34.74 | 0.566 |
| Tumor location | Upper/middle | 1.28 | 0.43-3.80 | 0.653 | 0.96 | 0.24-3.84 | 0.958 |
|  | lower/middle | 0.57 | 0.27-1.22 | 0.147 | 0.27 | 0.09-0.82 | 0.021* |
| Tumor grade | Well/moderately | 0.59 | 0.17-1.99 | 0.391 | 1.15 | 0.25-5.30 | 0.857 |
|  | Poorly/moderately | 1.69 | 0.81-3.53 | 0.158 | 2.32 | 0.78-6.87 | 0.128 |
| MetS | With/without | 1.86 | 0.86-4.00 | 0.114 | 2.38 | 0.75-7.54 | 0.141 |
| LncRNA-signature | High/low | 2.36 | 1.18-4.70 | 0.015* | 3.00 | 1.18-7.62 | 0.020* |
| BMI | Yes/no | 0.80 | 0.37-1.73 | 0.572 | — | — | — |
| Hyperglycemia | Yes/no | 1.47 | 0.72-2.99 | 0.288 | — | — | — |
| Hypertension | Yes/no | 2.00 | 1.00-3.99 | 0.049* | — | — | — |
| Triglycerides | Yes/no | 0.83 | 0.29-2.36 | 0.722 | — | — | — |
| HDL-C | Yes/no | 1.60 | 0.80-3.22 | 0.187 | — | — | — |
| LDL-C | Yes/no | 0.92 | 0.45-1.86 | 0.811 | — | — | — |
| Arrhythmia | Yes/no | 0.74 | 0.33-1.64 | 0.457 | — | — | — |
| Pneumonia | Yes/no | 0.51 | 0.07-3.74 | 0.508 | — | — | — |
| Anastomotic leak | Yes/no | 0.00 | 0.00-inf | 0.997 | — | — | — |

**p*<0.05, ***p*<0.01, ****p*<0.001

Association between six-lncRNA signature and recurrence free survival (RFS) in validation cohort (60 patients) in a univariate and multivariable analysis

|  |  |  | Univariable |  |  | Multivariable |  |
| --- | --- | --- | --- | --- | --- | --- | --- |
| Variable |  | Hazard ratio | 95% confidence interval | *p* | Hazard ratio | 95% confidence interval | *p* |
| Age | <50/50-59 | 1.45 | 0.52-4.09 | 0.479 | 1.29 | 0.33-5.05 | 0.717 |
|  | 60-69/50-59 | 0.67 | 0.26-1.73 | 0.407 | 0.84 | 0.22-3.25 | 0.802 |
|  | 70-79/50-59 | 0.85 | 0.18-3.93 | 0.830 | 1.19 | 0.11-12.76 | 0.886 |
|  | >80/50-59 | 1.29 | 0.16-10.26 | 0.809 | 0.85 | 0.07-10.91 | 0.902 |
| Gender | Female/male | 0.54 | 0.16-1.79 | 0.310 | ­— | — | — |
| Tobacco use | Yes/no | 1.63 | 0.71-3.75 | 0.250 | — | — | — |
| Alcohol use | Yes/no | 1.59 | 0.71-3.57 | 0.261 | — | — | — |
| Adjuvant therapy | Yes/no | 20.59 | 2.78-152.33 | 0.003** | 54.07 | 3.56-821.08 | 0.004** |
| T stage | T1/T3 | 0.49 | 0.07-3.63 | 0.483 | 64.08 | 0.76-5389.74 | 0.065 |
|  | T2/T3 | 0.85 | 0.25-2.86 | 0.791 | 5.05 | 0.29-87.36 | 0.265 |
|  | T4/T3 | 2.01 | 0.27-15.06 | 0.498 | 0.45 | 0.03-6.07 | 0.544 |
| N stage | N1/N0 | 3.3 | 1.24-8.82 | 0.017* | 0.07 | 0.00-2.64 | 0.151 |
|  | N2/N0 | 4.55 | 1.45-14.28 | 0.009** | 0.06 | 0.00-4.94 | 0.213 |
|  | N3/N0 | 11.5 | 0 2.22-59.52 | 0.003** | 0.47 | 0.01-20.59 | 0.693 |
| TNM stage | T1/T3 | 0.00 | 0.00-Inf | 0.997 | 0.00 | 0.00-Inf | 0.996 |
|  | T2/T3 | 2.72 | 1.21-6.14 | 0.015* | 10.10 | 0.31-327.54 | 0.192 |
| Tumor location | Upper/middle | 1.04 | 0.23-4.71 | 0.957 | 0.41 | 0.06-2.91 | 0.371 |
|  | lower/middle | 1.17 | 0.52-2.61 | 0.709 | 0.51 | 0.15-1.72 | 0.277 |
| Tumor grade | Well/moderately | 0.37 | 0.09-1.59 | 0.179 | 0.57 | 0.08-4.02 | 0.571 |
|  | Poorly/moderately | 0.72 | 0.28-1.81 | 0.482 | 0.83 | 0.19-3.53 | 0.798 |
| MetS | With/without | 1.80 | 0.75-4.30 | 0.186 | 1.32 | 0.42-4.16 | 0.635 |
| LncRNA-signature | High/low | 2.12 | 0.98-4.60 | 0.057 | 3.37 | 0.96-11.81 | 0.058 |
| BMI | Yes/no | 1.39 | 0.62-3.11 | 0.428 | — | — | — |
| Hyperglycemia | Yes/no | 1.50 | 0.68-3.32 | 0.316 | — | — | — |
| Hypertension | Yes/no | 2.09 | 0.95-4.59 | 0.065 | — | — | — |
| Triglycerides | Yes/no | 0.45 | 0.11-1.89 | 0.273 | — | — | — |
| HDL-C | Yes/no | 0.80 | 0.34-1.91 | 0.619 | — | — | — |
| LDL-C | Yes/no | 0.98 | 0.44-2.16 | 0.955 | — | — | — |
| Arrhythmia | Yes/no | 0.40 | 0.14-1.15 | 0.088 | — | — | — |
| Pneumonia | Yes/no | 1.46 | 0.34-6.19 | 0.608 | — | — | — |
| Anastomotic leak | Yes/no | 0.00 | 0.00-inf | 0.997 | — | — | — |

**p*<0.05, ***p*<0.01, ****p*<0.001
