# Supplementary material for: The Prognostic Significance of Metabolic Syndrome and a Related Six-lncRNA Signature in Esophageal Squamous Cell Carcinoma
Source: Front Oncol. 2020 Feb 18;10:61. doi: 10.3389/fonc.2020.00061 (PMC7040247; doi:10.3389/fonc.2020.00061)
Supplement: Supplementary file 5 [file Table_5.DOCX]

Supplement Table 5 Association between six-lncRNA signature and overall survival (OS) in 179 patients in a univariate and multivariable analysis

|  |  |  | Univariable |  |  | Multivariable |  |
| --- | --- | --- | --- | --- | --- | --- | --- |
| Variable |  | Hazard ratio | 95% confidence interval | p | Hazard ratio | 95% confidence interval | p |
| Age | <50/50-59 | 0.91 | 0.46-1.78 | 0.776 | 0.79 | 0.38-1.65 | 0.535 |
|  | 60-69/50-59 | 1.35 | 0.87-2.11 | 0.185 | 1.61 | 0.96-2.67 | 0.069 |
|  | 70-79/50-59 | 1.91 | 1.01-3.61 | 0.047* | 2.26 | 1.10-4.62 | 0.026* |
|  | >80/50-59 | 5.39 | 1.64-17.69 | 0.005** | 23.95 | 6.44-89.00 | 0.0001*** |
| Gender | Female/male | 1.28 | 0.80-2.05 | 0.306 | ­— | — | — |
| Tobacco use | Yes/no | 0.75 | 0.51-1.10 | 0.144 | — | — | — |
| Alcohol use | Yes/no | 0.86 | 0.59-1.27 | 0.457 | — | — | — |
| Adjuvant therapy | Yes/no | 1.93 | 1.22-3.04 | 0.004 | 1.52 | 0.89-2.59 | 0.125 |
| T stage | T1/T3 | 0.97 | 0.44-2.12 | 0.935 | 1.28 | 0.43-3.85 | 0.659 |
|  | T2/T3 | 1.05 | 0.60-1.82 | 0.870 | 1.29 | 0.65-2.54 | 0.468 |
|  | T4/T3 | 1.64 | 1.00-2.67 | 0.048* | 1.29 | 0.70-2.36 | 0.410 |
| N stage | N1/N0 | 2.04 | 1.31-3.18 | 0.001*** | 1.23 | 0.60-2.51 | 0.565 |
|  | N2/N0 | 2.05 | 1.14-3.70 | 0.017* | 0.88 | 0.34-2.25 | 0.784 |
|  | N3/N0 | 2.97 | 1.42-6.19 | 0.003** | 1.95 | 0.69-5.49 | 0.204 |
| TNM stage | T1/T2 | 0.56 | 0.17-1.82 | 0.336 | 0.58 | 0.12-2.74 | 0.489 |
|  | T3/T2 | 2.03 | 1.35-3.06 | 0.0006*** | 1.44 | 0.65-3.22 | 0.372 |
| Tumor location | Upper/middle | 1.47 | 0.83-2.59 | 0.186 | 1.41 | 0.72-2.77 | 0.316 |
|  | lower/middle | 0.88 | 0.57-1.35 | 0.561 | 0.65 | 0.40-1.06 | 0.082 |
| Tumor grade | Well/moderately | 0.99 | 0.57-1.70 | 0.961 | 1.06 | 0.56-2.01 | 0.853 |
|  | Poorly/moderately | 1.63 | 1.06-2.50 | 0.024* | 1.98 | 1.21-3.26 | 0.006** |
| MetS | With/without | 1.73 | 1.06-2.82 | 0.027* | 2.67 | 1.48-4.83 | 0.001** |
| LncRNA-signature | High/low | 3.08 | 2.06-4.59 | 0.0001*** | 3.97 | 2.47-6.36 | 0.0001*** |
| Arrhythmia | Yes/no | 1.12 | 0.73-1.72 | 0.608 | — | — | — |
| Pneumonia | Yes/no | 1.43 | 0.72-2.83 | 0.309 | — | — | — |
| Anastomotic leak | Yes/no | 1.30 | 0.60-2.80 | 0.503 | — | — | — |
| BMI | Yes/no | 1.30 | 0.86-1.96 | 0.220 | — | — | — |
| Hyperglycemia | Yes/no | 1.24 | 0.83-1.83 | 0.294 | — | — | — |
| Hypertension | Yes/no | 1.24 | 0.83-1.84 | 0.290 | — | — | — |
| Triglycerides | Yes/no | 1.11 | 0.65-1.89 | 0.701 | — | — | — |
| HDL-C | Yes/no | 0.95 | 0.59-1.54 | 0.848 | — | — | — |
| LDL-C | Yes/no | 1.07 | 0.72-1.59 | 0.752 | — | — | — |

**p*<0.05, ***p*<0.01, ****p*<0.001

Association between six-lncRNA signature and recurrence free survival(RFS) in 179 patients in a univariate and multivariable analysis

|  |  |  | Univariable |  |  | Multivariable |  |
| --- | --- | --- | --- | --- | --- | --- | --- |
| Variable |  | Hazard ratio | 95% confidence interval | *p* | Hazard ratio | 95% confidence interval | *p* |
| Age | <50/50-59 | 1.36 | 0.74-2.51 | 0.325 | 1.80 | 0.91-3.58 | 0.092 |
|  | 60-69/50-59 | 0.75 | 0.45-1.26 | 0.283 | 0.98 | 0.54-1.77 | 0.942 |
|  | 70-79/50-59 | 0.97 | 0.43-2.21 | 0.947 | 1.29 | 0.53-3.16 | 0.579 |
|  | >80/50-59 | 1.13 | 0.15-8.32 | 0.901 | 3.66 | 0.45-29.82 | 0.225 |
| Gender | Female/male | 0.75 | 0.41-1.39 | 0.359 | — | — | — |
| Tobacco use | Yes/no | 1.18 | 0.74-1.88 | 0.492 | — | — | — |
| Alcohol use | Yes/no | 1.31 | 0.83-2.06 | 0.247 | — | — | — |
| Adjuvant therapy | Yes/no | 8.50 | 3.69-19.55 | 0.0001*** | 6.49 | 2.71-15.54 | 0.0001*** |
| T stage | T1/T3 | 1.13 | 0.48-2.65 | 0.776 | 1.13 | 0.35-3.66 | 0.842 |
|  | T2/T3 | 0.80 | 0.39-1.64 | 0.548 | 1.13 | 0.48-2.69 | 0.778 |
|  | T4/T3 | 2.38 | 1.41-4.04 | 0.001** | 1.37 | 0.73-2.56 | 0.326 |
| N stage | N1/N0 | 2.51 | 1.47-4.29 | 0.0001*** | 0.96 | 0.42-2.19 | 0.919 |
|  | N2/N0 | 3.26 | 1.67-6.39 | 0.0001*** | 0.88 | 0.32-2.45 | 0.803 |
|  | N3/N0 | 6.56 | 3.08-14.00 | 0.0001*** | 2.55 | 0.81-8.07 | 0.110 |
| TNM stage | T1/T2 | 1.15 | 0.34-3.89 | 0.822 | 1.71 | 0.39-7.53 | 0.477 |
|  | T3/T2 | 3.67 | 2.18-6.18 | 0.0001*** | 2.40 | 0.95-6.08 | 0.06 |
| Tumor location | Upper/middle | 1.50 | 0.79-2.83 | 0.217 | 1.07 | 0.50-2.27 | 0.8634 |
|  | lower/middle | 0.85 | 0.52-1.39 | 0.513 | 0.61 | 0.35-1.07 | 0.082 |
| Tumor grade | Well/moderately | 0.76 | 0.39-1.46 | 0.403 | 0.85 | 0.41-1.78 | 0.666 |
|  | Poorly/moderately | 1.00 | 0.60-1.66 | 0.989 | 0.88 | 0.50-1.56 | 0.670 |
| MetS | With/without | 1.71 | 0.99-2.96 | 0.055 | 2.40 | 1.25-4.60 | 0.008** |
| LncRNA-signature | High/low | 3.22 | 2.03-5.10 | 0.0001*** | 3.23 | 1.95-5.35 | 0.0001*** |
| BMI | Yes/no | 1.24 | 0.77-2.00 | 0.373 | — | — | — |
| Hyperglycemia | Yes/no | 1.58 | 1.02-2.46 | 0.040* | — | — | — |
| Hypertension | Yes/no | 1.14 | 0.72-1.79 | 0.574 | — | — | — |
| Triglycerides | Yes/no | 1.04 | 0.57-1.93 | 0.888 | — | — | — |
| HDL-C | Yes/no | 0.68 | 0.38-1.24 | 0.211 | — | — | — |
| LDL-C | Yes/no | 0.93 | 0.59-1.48 | 0.759 | — | — | — |
| Arrhythmia | Yes/no | 0.99 | 0.59-1.66 | 0.981 | — | — | — |
| Pneumonia | Yes/no | 1.10 | 0.48-2.53 | 0.821 | — | — | — |
| Anastomotic leak | Yes/no | 1.03 | 0.42-2.54 | 0.952 | — | — | — |

**p*<0.05, ***p*<0.01, ****p*<0.001
