# Supplementary material for: The Prognostic Significance of Metabolic Syndrome and a Related Six-lncRNA Signature in Esophageal Squamous Cell Carcinoma
Source: Front Oncol. 2020 Feb 18;10:61. doi: 10.3389/fonc.2020.00061 (PMC7040247; doi:10.3389/fonc.2020.00061)
Supplement: Supplementary file 6 [file Table_6.DOCX]

Supplement Table 6 Association between lncRNA signature and overall survival (OS) in 80 patients from TCGA in a univariate and multivariable analysis

|  |  |  | Univariable |  |  | Multivariable |  |
| --- | --- | --- | --- | --- | --- | --- | --- |
| Variable |  | Hazard ratio | 95% confidence interval | p | Hazard ratio | 95% confidence interval | p |
| Age | <50/50-59 | 1.10 | 0.33-3.69 | 0.873 | 6.49 | 0.80-52.95 | 0.080 |
|  | 60-69/50-59 | 1.64 | 0.56-4.82 | 0.365 | 9.01 | 1.03-79.10 | 0.047 |
|  | 70-79/50-59 | 2.46 | 0.62-9.76 | 0.201 | 9.25 | 0.96-88.88 | 0.054 |
|  | >80/50-59 | 2.42 | 0.28-20.58 | 0.418 | 64.38 | 2.04-2027.13 | 0.018* |
| Gender | Female/male | 0.10 | 0.01-0.79 | 0.028* | 0.07 | 0.00-1.32 | 0.075 |
| Cigarettes | >2/<2 | 0.78 | 0.28-2.19 | 0.633 | 1.72 | 0.26-11.24 | 0.569 |
| Alcohol use | Yes/no | 3.13 | 0.73-13.44 | 0.125 | 539.25 | 6.09-47749.78 | 0.006** |
| T stage  N stage | T1/T3  T2/T3  T4/T3  N1/N0  N2/N0  N3/N0 | 1.11  1.29  3.40  2.07  3.85  6.11 | 0.30-4.15  0.49-3.41  0.70-16.47  0.82-5.25  1.03-14.43  0.74-50.67 | 0.874  0.602  0.127  0.123  0.045*  0.093 | 0.12  1.01  64.55  3.44  426.30  5792.13 | 0.00-6.26  0.13-8.06  1.90-2197.86  0.39-30.33  8.66-20984.13  8.02-4182235.98 | 0.297  0.989  0.021*  0.265  0.002**  0.009** |
| TNM stage | T1/T2 | 1.03 | 0.22-4.83 | 0.967 | 14.48 | 0.35-602.55 | 0.160 |
|  | T3/T2  T4/T2 | 1.96  5.38 | 0.74-5.20  1.82-15.96 | 0.176  0.002** | 0.10  78.44 | 0.00-3.37  2.30-2680.50 | 0.197  0.015* |
| Tumor location | Upper/lower | 0.00 | 0.00-inf | 0.997 | 0.00 | 0.00-inf | 0.998 |
|  | Middle/lower  NOS//lower | 1.25  0.47 | 0.52-3.02  0.06-3.63 | 0.614  0.468 | 1.32  2.08 | 0.19-9.12  0.06-76.07 | 0.778  0.689 |
| LncRNA-signature | High/low | 2.52 | 1.06-6.01 | 0.036* | 5.37 | 1.02-28.41 | 0.047* |
| BMI | >25/<25 | 0.86 | 0.31-2.35 | 0.764 | 0.91 | 0.07-11.12 | 0.941 |
|  |  |  |  |  |  |  |  |

**p*<0.05, ***p*<0.01, ****p*<0.001

Association between lncRNA signature and recurrence free survival (RFS) in 80 patients from TCGA in a univariate and multivariable analysis

|  |  |  | Univariable |  |  | Multivariable |  |
| --- | --- | --- | --- | --- | --- | --- | --- |
| Variable |  | Hazard ratio | 95% confidence interval | *p* | Hazard ratio | 95% confidence interval | *p* |
| Age | <50/50-59 | 1.67 | 0.62-4.51 | 0.308 | 1.16 | 0.33-4.11 | 0.820 |
|  | 60-69/50-59 | 1.51 | 0.58-3.93 | 0.402 | 1.01 | 0.29-3.54 | 0.993 |
|  | 70-79/50-59 | 1.30 | 0.35-4.86 | 0.699 | 0.58 | 0.10-3.36 | 0.541 |
|  | >80/50-59 | 1.25 | 0.16-9.99 | 0.831 | 0.80 | 0.06-10.67 | 0.862 |
| Gender | Female/male | 0.42 | 0.13-1.40 | 0.156 | 0.37 | 0.05-2.82 | 0.340 |
| Cigarettes | Yes/no | 1.54 | 0.60-3.97 | 0.374 | 1.26 | 0.28-5.53 | 0.763 |
| Alcohol use | Yes/no | 1.78 | 0.62-5.13 | 0.286 | 1.24 | 0.31-4.96 | 0.762 |
| T stage | T1/T3 | 0.22 | 0.03-1.66 | 0.141 | 1.60 | 0.06-42.79 | 0.778 |
|  | T2/T3 | 0.74 | 0.33-1.66 | 0.468 | 0.47 | 0.15-1.51 | 0.205 |
|  | T4/T3 | 0.00 | 0.00-Inf | 0.997 | 0.00 | 0.00-Inf | 0.998 |
| N stage | N1/N0 | 0.47 | 0.18-1.27 | 0.137 | 0.05 | 0.00-1.28 | 0.070 |
|  | N2/N0 | 0.50 | 0.07-3.76 | 0.502 | 0.03 | 0.00-1.50 | 0.079 |
|  | N3/N0 | 1.78 | 0.24-13.41 | 0.574 | 0.03 | 0.00-3.33 | 0.144 |
| TNM stage | T1/T3 | 0.31 | 0.04-2.31 | 0.251 | 0.36 | 0.03-5.00 | 0.447 |
|  | T2/T3 | 0.78 | 0.31-1.96 | 0.603 | 8.96 | 0.34-233.42 | 0.187 |
|  | T4/T3 | 1.07 | 0.25-4.64 | 0.923 | 0.00 | 0.00-Inf | 0.998 |
| Tumor location | Upper/middle | 2.06 | 0.45-9.51 | 0.354 | 6.90 | 0.84-56.57 | 0.071 |
|  | lower/middle | 1.76 | 0.79-3.95 | 0.169 | 1.45 | 0.41-5.14 | 0.565 |
|  | NOS//lower | 0.44 | 0.06-3.41 | 0.429 | 0.44 | 0.04-5.05 | 0.508 |
| LncRNA-signature | High/low | 2.08 | 0.97-4.46 | 0.060 | 3.47 | 1.04-11.62 | 0.043* |
| BMI | Yes/no | 0.69 | 0.26-1.84 | 0.463 | 0.61 | 0.09-4.30 | 0.622 |
|  |  |  |  |  |  |  |  |

**p*<0.05, ***p*<0.01, ****p*<0.001
