# Supplementary figures and images for: The Prognostic Significance of Metabolic Syndrome and a Related Six-lncRNA Signature in Esophageal Squamous Cell Carcinoma
Source: Front Oncol. 2020 Feb 18;10:61. doi: 10.3389/fonc.2020.00061 (PMC7040247; doi:10.3389/fonc.2020.00061)

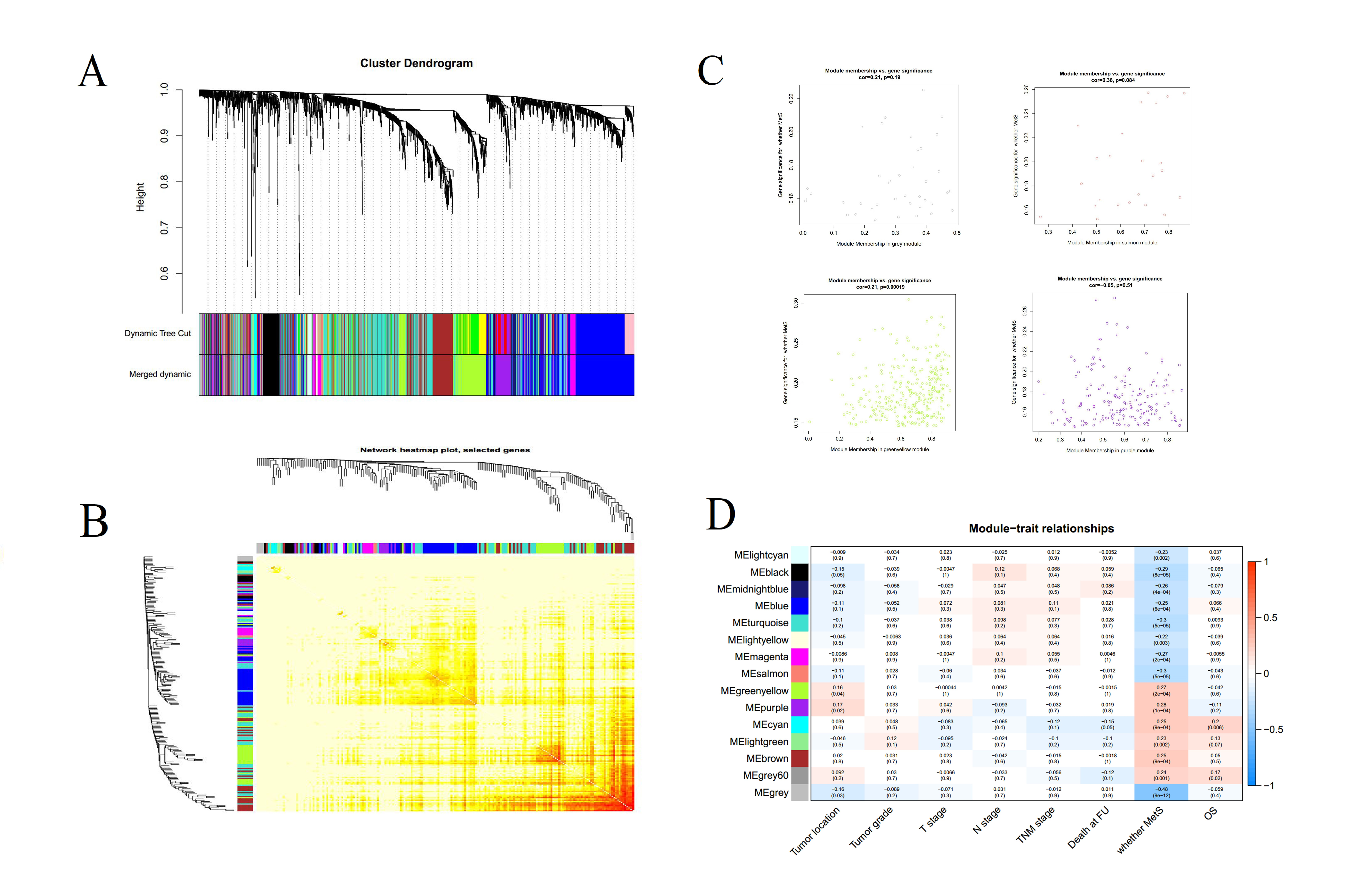

Supplement: Supplementary file 7 [file Image_1.TIF]

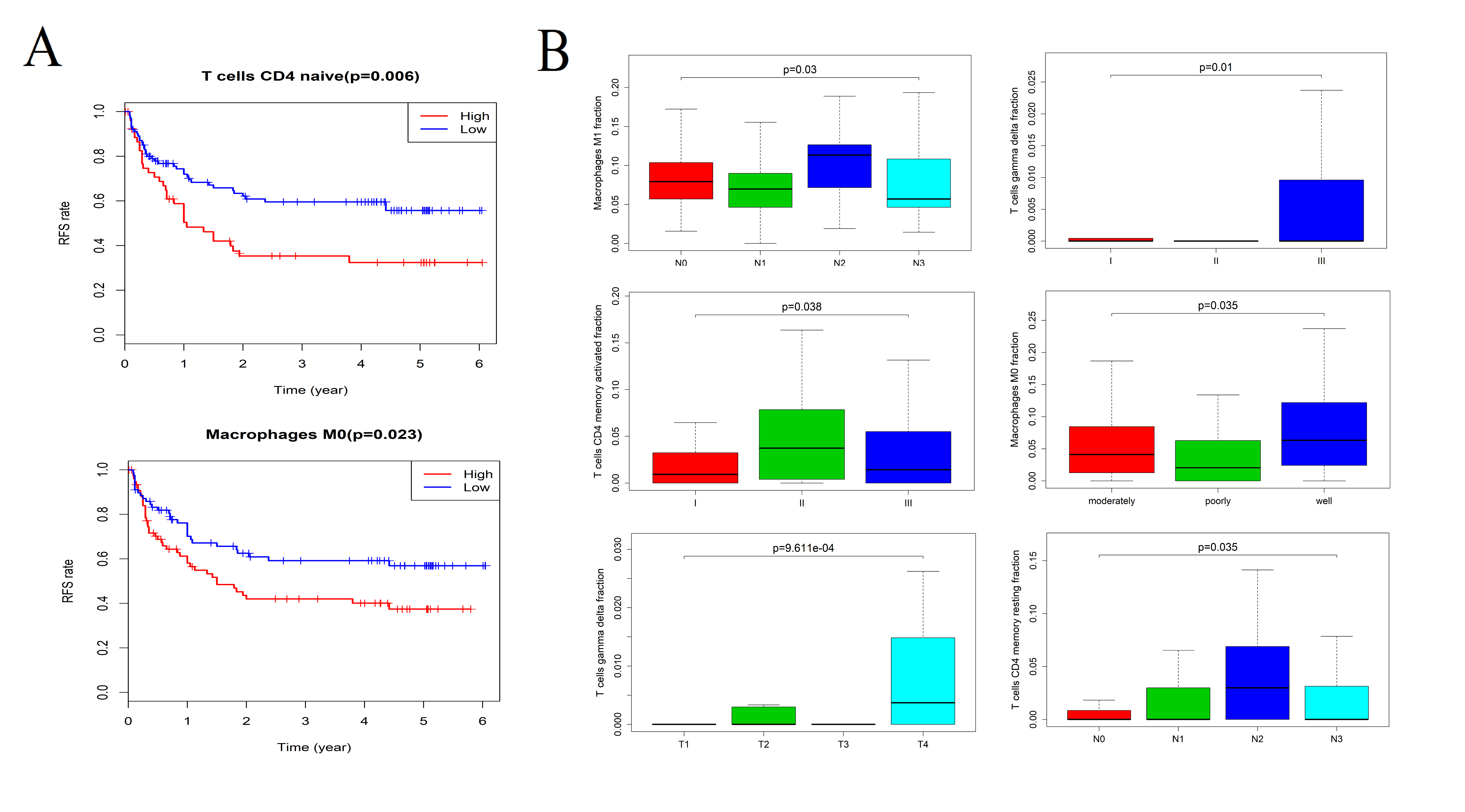

Supplement: Supplementary file 8 [file Image_2.TIF]

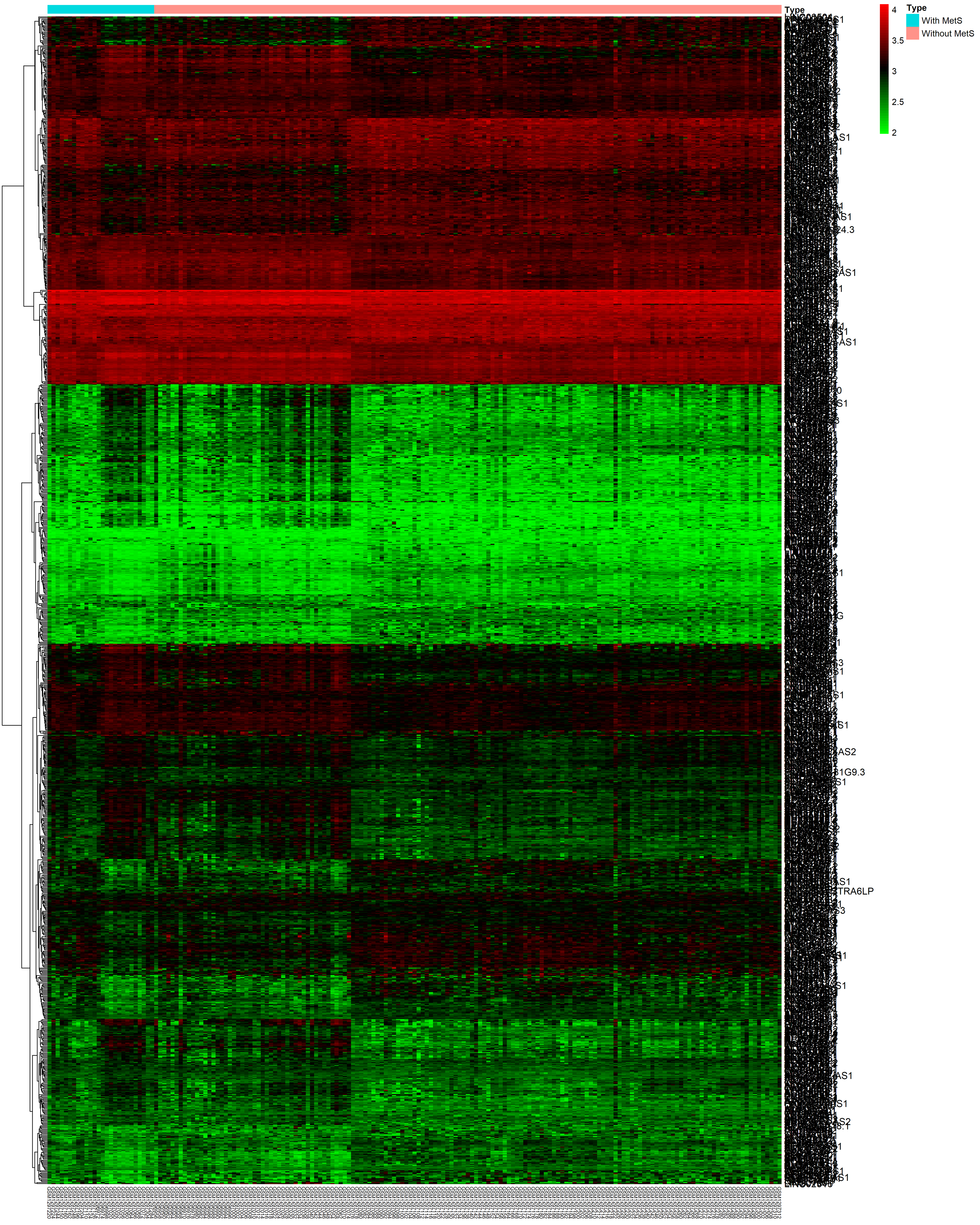

Supplement: Supplementary file 10 [file Image_4.TIF]

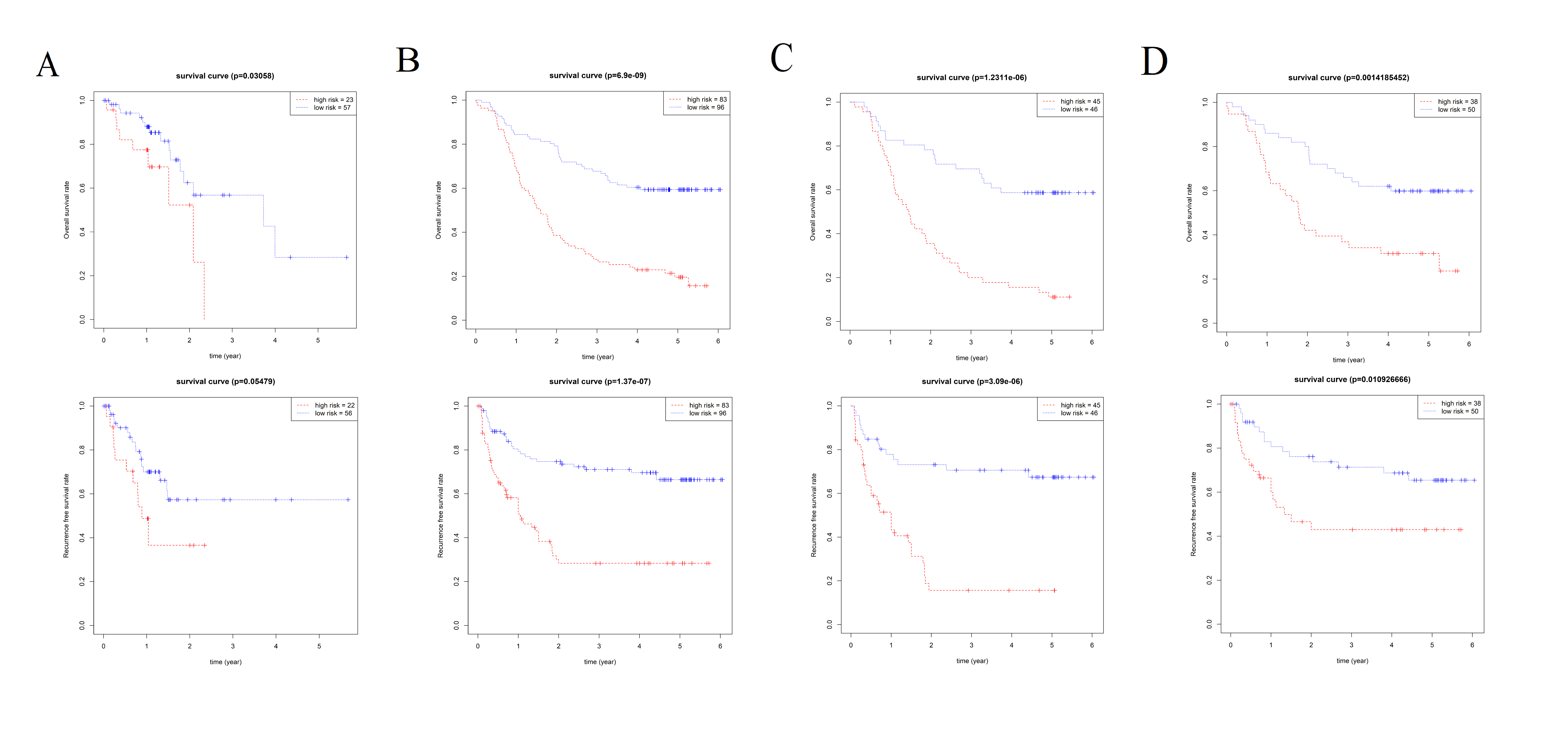

Supplement: Supplementary file 11 [file Image_5.TIF]

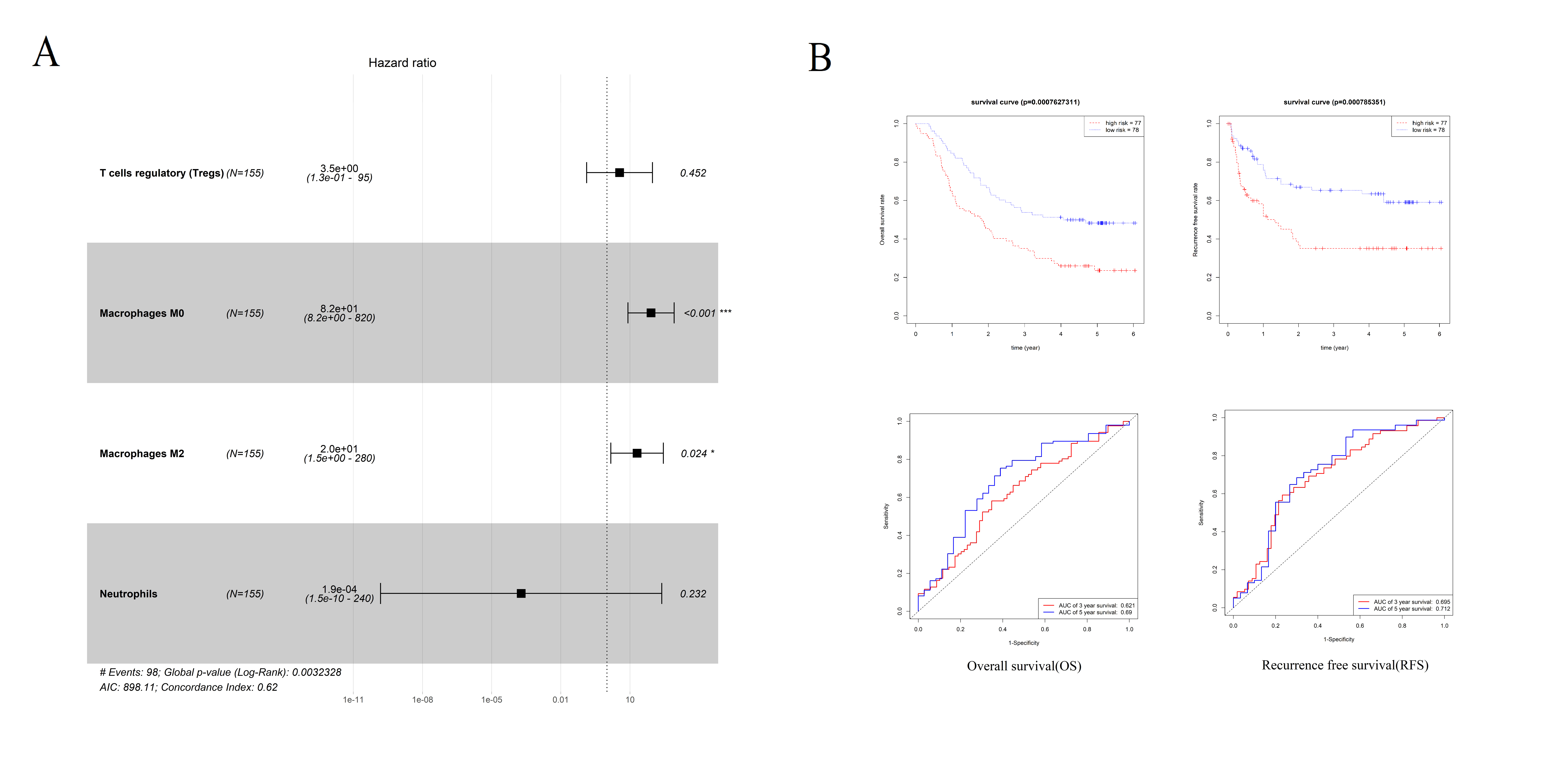

Supplement: Supplementary file 12 [file Image_6.TIF]

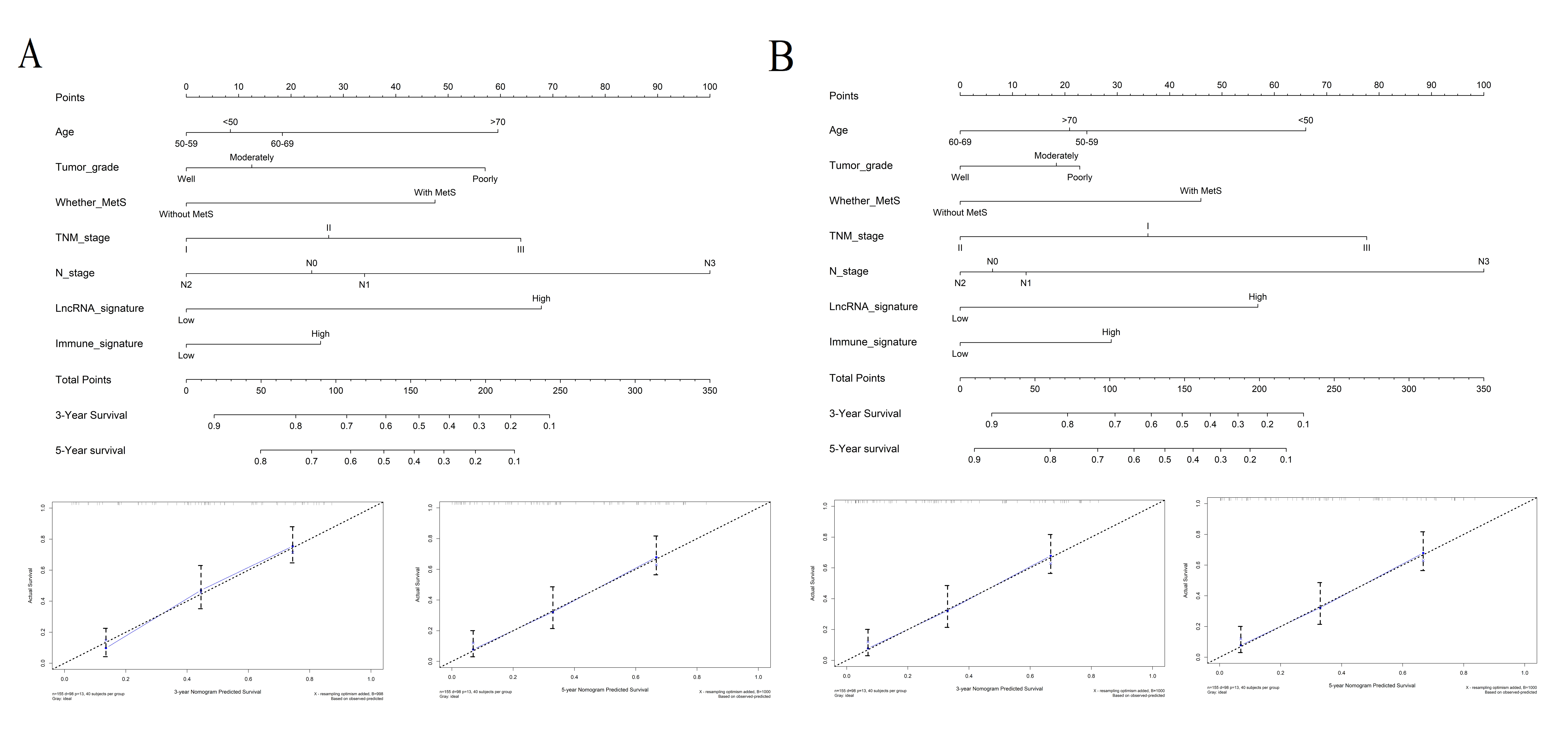

Supplement: Supplementary file 13 [file Image_7.TIF]

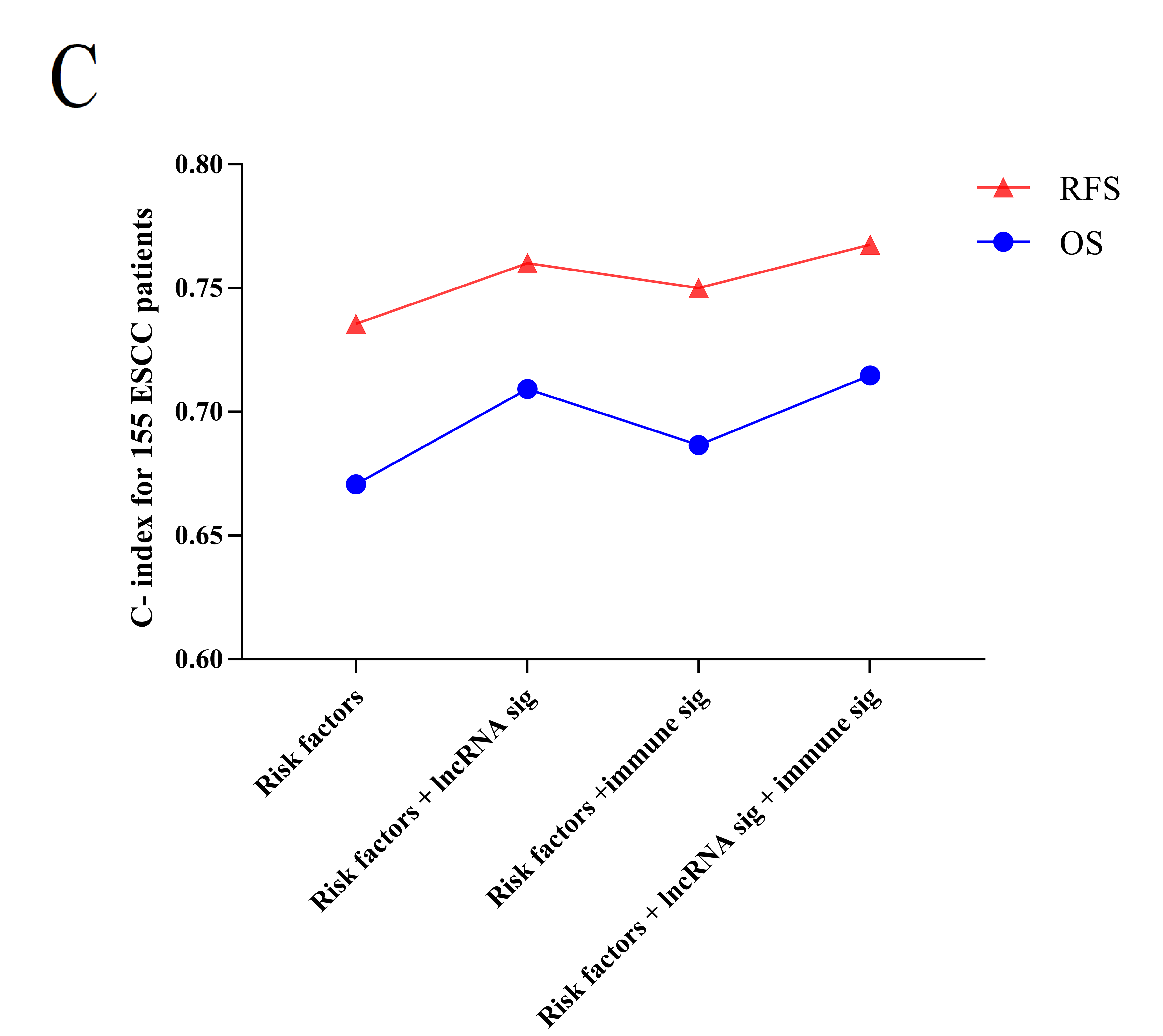

Supplement: Supplementary file 14 [file Image_8.TIF]
