# Supplementary material for: The Prognostic Significance of Metabolic Syndrome and a Related Six-lncRNA Signature in Esophageal Squamous Cell Carcinoma
Source: Front Oncol. 2020 Feb 18;10:61. doi: 10.3389/fonc.2020.00061 (PMC7040247; doi:10.3389/fonc.2020.00061)
Supplement: Supplementary file 15 [file Data_Sheet_1.docx]

**Supplement image legends**

**Image 1: The selection of mRNA module using WGCNA**

The merged dynamic of lncRNA module(A), network heatmap plot (B), four module membership with gene significant (C) and the module trait relationship (D).

**Image 2: The relationship between immune infiltration and clinical status**

(A) The infiltration of T cells CD4 naive and infiltration of M0 macrophages were all associated with RFS

(B) The clinical status was significant associated with immune infiltration (*p* < 0.05)

**Image 3: The volcano plot for difference lncRNA.**

**Image 4: The heatmap for the difference lncRNA.**

**Image 5: The verification of six-lncRNA signature in 80 patients from TCGA, 179 patients and random grouping**

The Kaplan-Meier survival curve analysis was shown in 80 ESCC patients from TCGA (A), 179 ESCC patients (B), 91 patients (C) and 88 patients (D) from random grouping

**Image 6: The forest plot and identification of the immune infiltration signature**

1. The forest plot of four-immune infiltration signature
2. The OS and RFS for survival status and ROC curves of the four-immune infiltration signature.

**Image 7: The establishment of clinical predictive model by immune infiltration signature**

The prognostic nomogram was established by Age, Tumor grade, MetS, TNM stage, N stage, lncRNA signature and immune signature for OS(A) and RFS(B), the calibration curves for the nomogram for 3- and 5- year were also shown.

**Image 8: The C-index were calculated for clinical predictive model.**
